# Granular Ionogel Particle Inks for 3D Printed Tough and Stretchable Ionotronics

**DOI:** 10.34133/research.0104

**Published:** 2023-06-07

**Authors:** Yuan Yao, Yue Hui, Zhenhua Wang, Hehao Chen, Heng Zhu, Nanjia Zhou

**Affiliations:** ^1^ Key Laboratory of 3D Micro/Nano Fabrication and Characterization of Zhejiang Province, School of Engineering, Westlake University, Hangzhou 310024, Zhejiang Province, China.; ^2^ Institute of Advanced Technology, Westlake Institute for Advanced Study, Hangzhou 310024, Zhejiang Province, China.; ^3^ School of Chemical Engineering and Advanced Materials, the University of Adelaide, Adelaide 5005, South Australia, Australia.; ^4^ State Key Laboratory of Fluid Power & Mechatronic System, Key Laboratory of Soft Machines and Smart Devices of Zhejiang Province, Center for X-Mechanics, Department of Engineering Mechanics, Zhejiang University, Hangzhou 310027, China.

## Abstract

Ionogels have garnered great attention as promising soft conducting materials for the fabrication of flexible energy storage devices, soft actuators, and ionotronics. However, the leakage of the ionic liquids, weak mechanical strength, and poor manufacturability have greatly limited their reliability and applications. Here, we propose a new ionogel synthesis strategy by utilizing granular zwitterionic microparticles to stabilize ionic liquids. The ionic liquids swell the microparticles and physically crosslink microparticles via either electronic interaction or hydrogen bonding. Further introducing a photocurable acrylic monomer enables the fabrication of double-network (DN) ionogels with high stretchability (>600%) and ultrahigh toughness (fracture energy > 10 kJ/m^2^). The synthesized ionogels exhibit a wide working temperature of −60 to 90 °C. By tuning the crosslinking density of microparticles and physical crosslinking strength of ionogels, we synthesize DN ionogel inks and print them into three-dimensional (3D) motifs. Several ionogel-based ionotronics are 3D printed as demonstrations, including strain gauges, humidity sensors, and ionic skins made of capacitive touch sensor arrays. Via covalently linking ionogels with silicone elastomers, we integrate the ionogel sensors onto pneumatic soft actuators and demonstrate their capacities in sensing large deformation. As our last demonstration, multimaterial direct ink writing is harnessed to fabricate highly stretchable and durable alternating-current electroluminescent devices with arbitrary structures. Our printable granular ionogel ink represents a versatile platform for the future manufacturing of ionotronics.

## Introduction

Gel matters are polymer networks that are solvated by liquid solvents. These liquids provide gels with ionic conductivity as well as excellent mechanical stretchability [1,2], enabling new applications in energy storage [3,4], soft robotics [5], wearable electronics [6–8], and biomedical engineering [9–12]. Based on different solvent types, gels are categorized as hydrogels, organogels, and ionogels. Among them, hydrogels and organogels have been extensively investigated due to their tunable mechanical strength [13], high stretchability [14], and manufacturability [15–17]. However, the rapid evaporation of solvents even at room temperature and the poor anti-freezing nature of water and organic solvents have prevented them from widespread applications. The incorporation of humectants such as hydratable salts [18] and glycerol analogues [19,20] or sealing gels with hydrophobic elastomers [21] have been shown to retard but not prevent dehydration or solvent evaporation completely. In contrast, ionogels, with the solvent component being ionic liquids (ILs), exhibit extraordinary anti-drying properties and excellent thermal stability [22,23], thanks to their inherent non-volatile, low melting point, and high thermal stability nature [1,24]. These unique features render ionogels promising candidates for the fabrication of soft ionotronic devices.

To date, ionogels are generally fabricated via in situ polymerization of gelators in ILs [23,25], polymerization of IL-based monomers [26], or swelling bulk polymeric matrices in ILs [27]. For the manufacturing of ionotronics to meet the diverse shape and material requirements demanded for emerging electronic applications [2], high resolution [28], high design freedom [29], and cost-effective manufacturing strategies are highly desirable. Recently, additive approaches such as direct ink writing (DIW) [22,30] have been employed to fabricate ionogels on demand. Yet, these demonstrations are limited to two-dimensional (2D) or 2.5D configurations and are insufficient for three-dimensional (3D) architectures with either high aspect ratios or spanning structures, mainly because of the unsatisfied rheological behaviors of the precursor-based (sol-like) ionogel inks [22,30,31]. With a generalized material synthetic strategy and tunable rheological behaviors, one can greatly expand the shape complexity and the functionalities of printed ionotronics, without the need of adding rheological modifiers or involving sophisticated dynamic crosslinking chemistry.

Gels made from granular microgel particles are intrinsically viscoelastic fluids, which can transform from their solid to liquid phases at high shear force and recover at rest [32], appearing to be a promising candidate for creating direct ink writable inks. Yang et al. [33] formulated a direct ink writable acrylic amide swollen poly(2-acrylamido-2-methyl-1-propanesulfonic sodium microgel particle ink. Via the incorporation of nanoclays that function both as rheological modifiers and as physical crosslinkers, the printed hydrogels exhibit both high stretchability and fracture toughness (~10 kJ/m^2^). The printed structures are also self-supportive even before curing. However, the hydrolysis of nanoclays when exposed to moisture in the atmosphere over time causes collapse of the printed architectures. By using jammed polymeric microgel particles that are swollen by acrylic monomers, 3D printable double-network (DN) hydrogel inks can be created without the need of extra rheological modifiers [34,35]. After curing, the interpenetrated polymer–microparticle networks can provide the printed structures with high toughness, but they are non-ideal for high stretchability due to the lack of a dynamic energy dissipation network. Although the physically crosslinked networks (dynamic energy dissipation network) can be post-formed by immersing the printed structures into metal ion-containing solutions, the enormous swelling of the prints is inevitable [36,37].

**Fig. 1.**
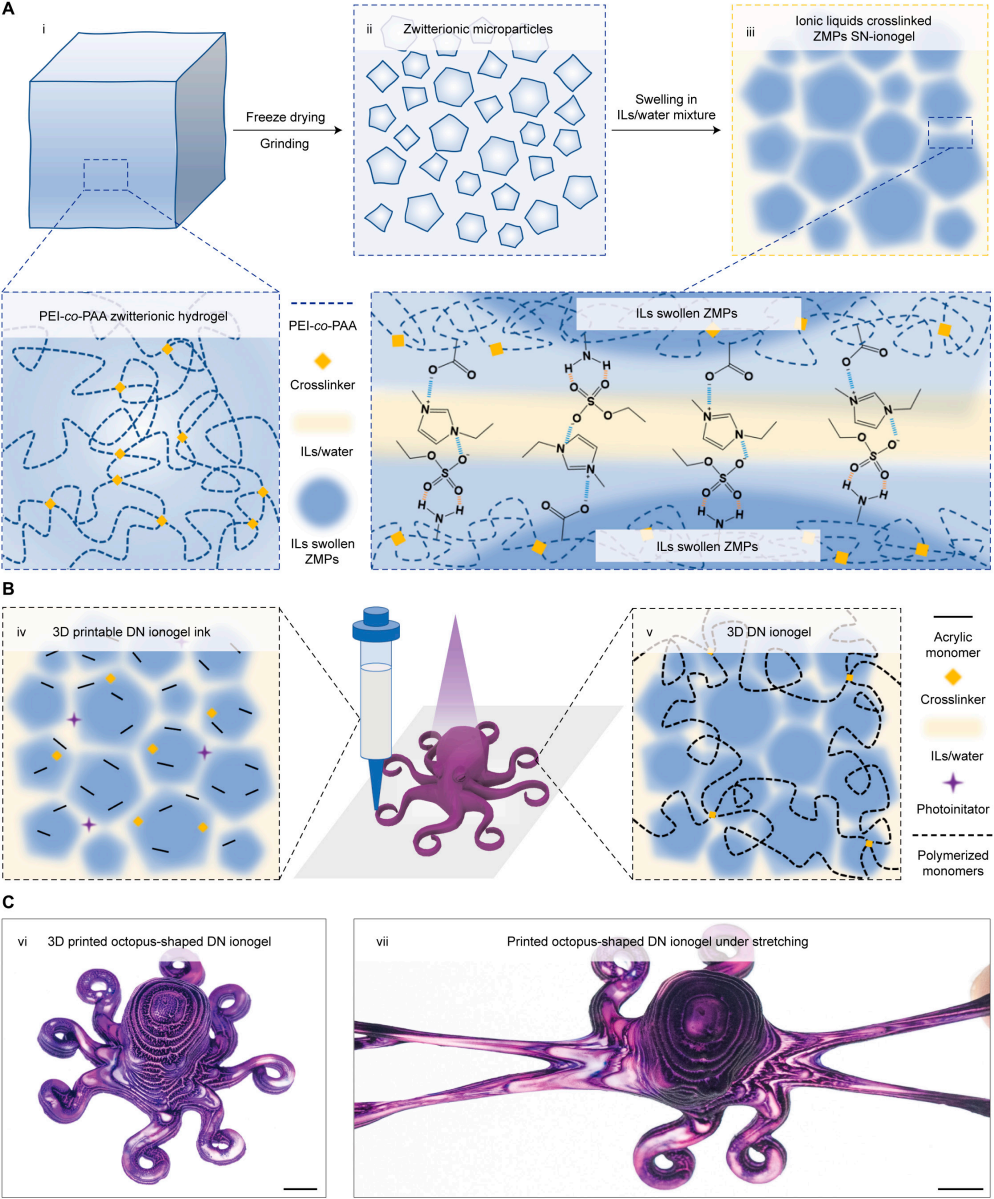
Synthetic strategy of the direct ink writable DN ionogel inks. (A) Schematic representation of the synthetic strategy of ZMP-stabilized ionogels. (i) Synthesis of PEI-*co*-PAA zwitterionic hydrogel, (ii) grinding freeze-dried zwitterionic hydrogel into microparticles, (iii) swelling ZMPs in ILs forming the IL-crosslinked ZMP SN-ionogel. (B) Schematic representation of the fabrication of direct ink writable DN ionogels into 3D motifs. (iv) Addition of an acrylic-based monomer, a crosslinker, and a photoinitiator into the IL-crosslinked ZMP ionogel fabricating 3D printable DN ionogel ink. (v) Curing printed ionogel inks to form stretchable and tough DN 3D ionogels. (C) The 3D printed stretchable and tough DN ionogels. (vi) 3D printed octopus-shaped DN ionogel dyed with purple color. (vii) The printed octopus-shaped DN ionogel under stretching (scale bar = 10 mm).

Here, we developed a generalized approach to synthesize DN ionogel inks for direct ink writable printed ionotronics with high stretchability and toughness (Fig. 1). The granular microparticles that possess high IL affinity are first synthesized and subsequently swollen by appropriate amount of ILs. The DN ionogel ink is then synthesized by introducing polymerizable acrylic-based monomers, crosslinkers, and photoinitiators into the IL-swollen zwitterionic microparticles (ZMPs). The strong physically crosslinked networks are formed by tuning the ratio of ILs and ZMPs, while the covalently crosslinked networks are established by in situ photocuring of acrylic monomers. The resulting ionogels not only exhibit high stretchability (>600%) and ultrahigh toughness (fracture energy > 10 kJ/m^2^), but also possess a wide working temperature of ~−60 to ~90 °C. The printability of the DN ionogel inks is further enabled by adjusting the crosslinking density of ZMPs and precisely controlling the dynamic crosslinking strength. Various 3D ionogel motifs are printed, including structures with high aspect ratios and self-spanning features. We further printed several ionogel-based ionotronics including resistive strain gauges, humidity sensors, and capacitive ionic skins. Via covalently bonding ionogels with silicone elastomers, we integrated the ionogel sensors onto pneumatic soft actuators and demonstrate its capability for large deformation sensing. As our last demonstration, multimaterial direct ink writable is harnessed to fabricate highly stretchable and durable alternating-current electroluminescent (ACEL) devices with arbitrary structures. Overall, our generalized strategy for formulating microparticle-based ionogel inks for direct ink writable opens new avenues for the future manufacturing of ionotronics.

## Results

The jammed granular hydrogel microparticles possessing yield-stress fluid-like rheological behaviors are commonly used in the field of additive manufacturing. For example, they can be used as the supporting matrix for embedded 3D printing [38–40], providing suitable cell culture environment for the fabrication of artificial tissues [41,42]. They can also function as basic units for constructing soft actuators [34]. By replacing water with ILs and using polymeric microparticles with high IL affinity, we adopt the granular microparticle strategy to formulate direct ink writable ionogel inks. We use 1-ethyl-3-methylimidazolium ethyl sulfate ([EMIM][EtOSO_3_]) as our model ILs. To start, the polymeric microparticles that can efficiently stabilize ILs need to be carefully designed with functional groups that possess strong IL affinities. Here, we synthesize a zwitterionic copolymer via graft copolymerization of an anionic monomer, acrylic acid (AAc), with a highly branched synthetic polymer with numerous cationic moieties, polyethyleneimine (PEI), leading to poly(acrylic acid)-*co*-polyethyleneimine (PAA-*co*-PEI) copolymers. Specifically, the branched PEI is first dissolved in 40 ml of deionized water, followed by the addition of a certain amount of AAc monomers and *N,N′*-methylenebisacrylamide (MBAA). The mixture is degassed in N_2_ for 30 min, and then heated to 80 °C to initiate the reaction. At 80 °C, the peroxide initiators (H_2_O_2_) can generate free radicals on amine groups from PEIs, which initiate the graft copolymerization of AAc monomers [43]. Besides, the hydroxyl radicals (generated by H_2_O_2_) in water can also initiate the homopolymerization of AAc monomers. The mixtures can gradually transform from solution to hydrogel in 1 h (in the first hour, the monomer conversion is already 85% to 90%). To achieve a higher monomer conversion (>95%), the reaction is allowed to last for 4 h. The Fourier transform infrared (FTIR) spectrum of the zwitterionic hydrogel is further investigated. As shown in Fig. S1, the N-H and C-N stretching peaks at ~3,430 and ~1,166 cm^−1^ indicate the presence of PEIs, while the C=O stretching peak at ~1,720 cm^−1^ from AAc moieties confirms that PAA has been successfully grafted onto PEIs. Through varying the monomer feeding ratios, we fine-tune the stoichiometric ratio of cationic to anionic moieties (PEI to AAc) ranging from 0.1:1 to 0.3:1. We next monitor the swelling behavior of the zwitterionic copolymer over a wide pH range. The zwitterionic copolymer maintains its weight in the pH range from 3 to 11 but swells enormously at pH 1 and pH 13 (Fig. S2A). The zwitterionic copolymer also exhibits an “anti-polyelectrolyte effect” in ILs/water mixture solutions (salts containing solutions) (Fig. S2B). Figure S3 illustrates the synthetic process of the ZMPs, in which the as-synthesized zwitterionic copolymers are first dialyzed to neutral pH, followed by a freeze-drying process to remove the water molecules. Thereafter, the copolymers are ground and ball-milled to form micron-sized particles (Fig. S4).

**Fig. 2.**
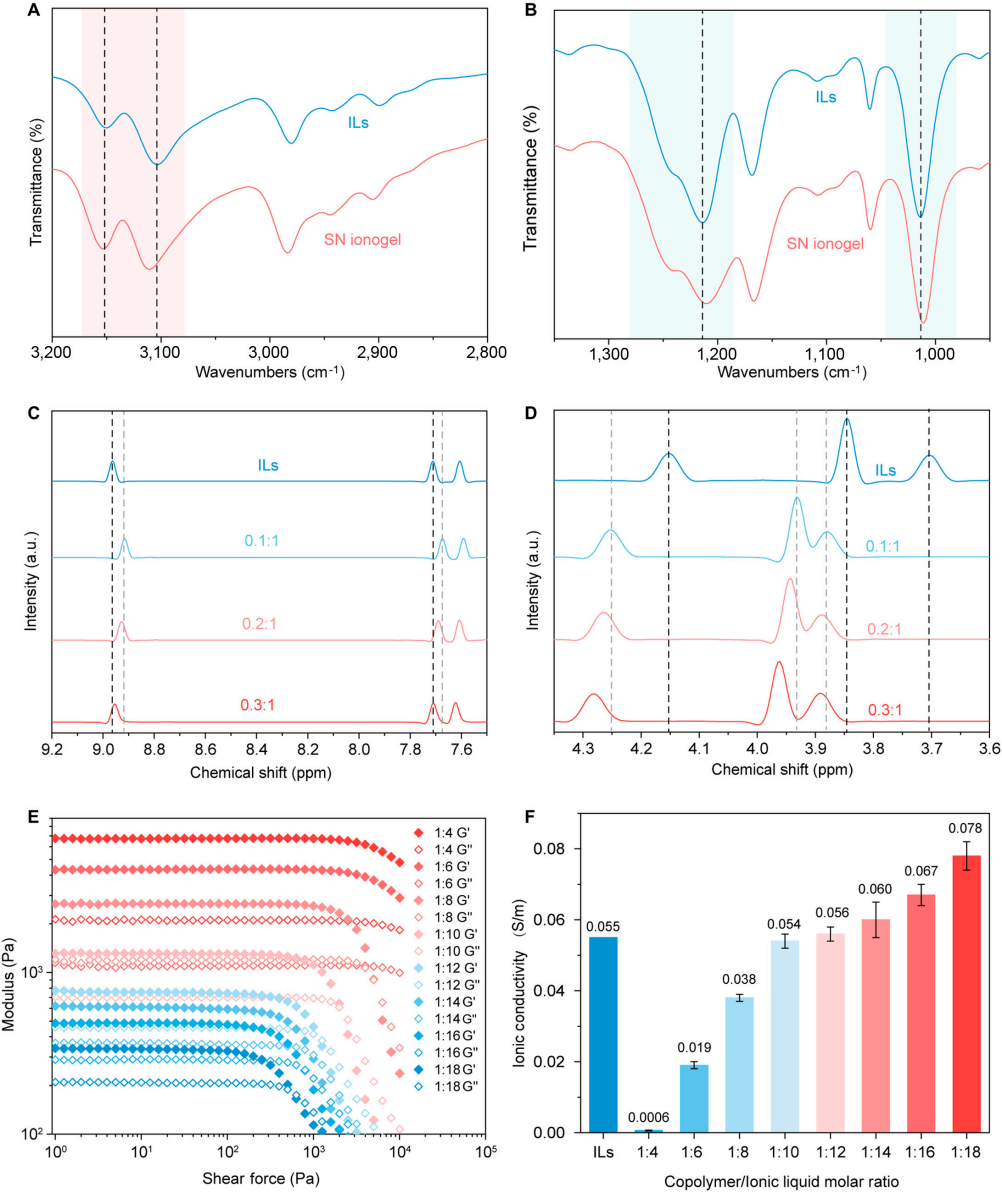
Molecular interaction between zwitterionic microparticles and ionic liquid. The FTIR spectra of native ionic liquid and the SN ionogel in the region of (A) 3,200 to 2,800 cm^−1^ and in the region of (B) 1,350 to 950 cm^−1^. The ^1^H-SSNMR spectra of ionic liquid and SN ionogels with different PEI-to-PAA ratio ranging from 0.1:1 to 0.3:1 in the region of (C) 9.2 to 7.5 ppm and (D) 4.35 to 3.6 ppm. (E) Storage (*G*′) and loss (*G*′) moduli as a function of shear stress of ionogels with different ionic liquid-to-ZMP weight ratios. (F) The ionic conductivity of ionogels with varying amounts of ionic liquid.

Next, we investigate the IL affinity of ZMPs with varying PEI-to-PAA ratios. A physically crosslinked single-network ionogel (SN-ionogel) is first prepared by swelling the ZMPs with ILs at a weight ratio of 1:4. The FTIR spectra of SN-ionogel are performed to investigate the interaction between ZMPs and ILs (Fig. 2A and B). The blue shift of the characteristic peaks of cationic EMIM and the red shift of the characteristic peaks of anionic EtOSO_3_ molecules indicate that the strong electronic interactions and hydrogen bonding are formed between ZMPs and ILs. Solid-state ^1^H-NMR is also adopted to investigate the IL affinity of ZMPs with varying PEI-to-PAA ratios. Figure 2C shows that increasing PAA content gradually shifts the peaks of the aromatic hydrogens from EMIM molecules to high field, indicating that a strong electronic interaction is formed between the carboxylic acid groups from ZMPs and the protonated tertiary amines in EMIM molecules. Meanwhile, the characteristic peaks of the aliphatic hydrogens from the negatively charged EtOSO_3_ molecules are downshifted with increasing of PEI content (Fig. 2D), indicating that the involvement of more amine moieties strengthens the interactions between ZMPs and EtOSO_3_ molecules. Among three different ratios, the ZMPs with a PEI-to-PAA ratio of 0.2:1 exhibits the highest affinity with both cationic and anionic molecules in ILs, and thus, it is chosen to fabricate ionogels in the following studies.

We then examine the IL stabilization capacity of the ZMPs. A series of SN-ionogels are produced by swelling ZMPs with different weight ratios of ILs ranging from 1:4 to 1:18. Figure 2E shows the rheological behaviors of the SN ionogels with varying IL weight ratios. With a ZMP-to-IL ratio below 1:8, the ionogel barely yields, even at an extremely high shear stress of ~1 × 10^4^ Pa. Incorporating more ILs weakens the interaction between ZMPs and IL, resulting in the appearance of a yield point. When the ZMP-to-IL weight ratio reaches 1:16, the loss modulus of the ionogel exceeds its storage modulus, indicating that the ILs act as solvent dispersing the ZMPs. To further ensure that the ILs can be stabilized in the ZMPs, we adopt a squeeze test. The SN ionogels with a ZMP-to-IL weight ratio below 1:10 exhibits high ILs stabilities. After squeezing ionogels, no leakage of ILs is observed (Fig. S5a), while the SN ionogels with a ZMP-to-IL weight ratio above 1:12 fail to maintain the ILs during squeezing. We also apply the squeeze test at a humid environment (humidity ~90%). Only the SN ionogels with a ZMP-to-IL weight ratio of 1:8 or below can efficiently stabilize the ILs (Fig. S5b). Next, the ionic conductivity of the ionogels is measured to verify whether the formation of strong molecular interaction compromises their ionic conductivity (Fig. 1F). When incorporating a small amount of ILs (ZMP-to-IL weight ratio below 1:4), the strong molecular interactions between ZMPs and IL reduce the mobilities of charge carriers in ILs, rendering ionogels with lower ionic conductivity compared to native ILs. The ionogel with a ZMP-to-IL weight ratio of 1:8 shows not only comparable ionic conductivity, but also strong ILs stabilization capacity under external pressure; thus, it was used to fabricate DN ionogel inks in the following studies.

**Fig. 3.**
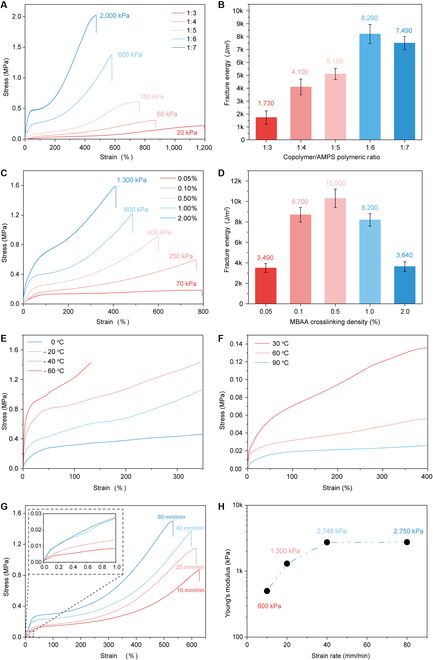
Fabrication of mechanically robust double-network ionogel ink. (A) The stress–strain curves of DN ionogels with different ZMP-to-AMPS weight ratios. (B) The fracture energy of DN ionogels with different ZMP-to-AMPS weight ratios. (C) The stress–strain curves of DN ionogels with different crosslinking densities. (D) The fracture energy of DN ionogels with different MBAA crosslinking densities. (E) The low (−60 to 0 °C) and (F) high (30 to 90 °C) temperature DMA test of DN ionogels. (G) The stress–strain curve of DN ionogel at varied strain rates. (H) The moduli of DN ionogels at different strain rates.

The ionogels are typically prepared by swelling a covalently crosslinked homo/co-polymer or in situ polymerization of acrylic-based monomers in ILs. Like water molecules in hydrogels, the ILs play the role as solvents to soften polymer chains, providing the covalently crosslinked polymer networks with high stretchability. However, the solvation effect of the ILs endows the ionogels with high stretchability but low toughness due to the lack of an energy dissipating network. Here, the mechanically robust ionogel is designed to possess a dynamically crosslinked network to dissipate energy while concurrently having a covalently crosslinked network to provide stretchability. Through varying the ZMP-to-IL ratios, we create a series of SN ionogels with strong intermolecular interactions between ILs and ZMPs. The ILs here act as a physical crosslinker, dynamically binding the ZMPs through either electronic interaction or hydrogen bonds, providing ionogels with sufficient energy dissipating networks. The covalently crosslinked networks are then introduced through the addition of a photocurable monomer 2-acrylamido-2-methylpropane sulfonic acid (AMPS) and a crosslinker MBAA. We first vary the polymeric ratio of ZMPs and AMPS from 1:3 to 1:7. By increasing the amount of the covalent proportion, the modulus of DN ionogels is increased from 20 kPa to 2 MPa. Meanwhile, the elongation at break of DN ionogels decreases from 1,200% to 490% (Fig. 3A). The fracture energy of the DN ionogels is then measured using the pure shear test [44]; the maximum fracture energy is determined as ~8,200 J/m^2^ at a ZMP-to-AMPS ratio of 1:6 (Fig. 3B and Fig. S6A), which is higher than most reported ionogels [27]. We further tune the crosslinking density of the covalent networks by varying the proportions of MBAA from 0.05% to 2%. The decrease of the crosslinking density promotes the elongation at break from 400% to 800% and results in reduced moduli of DN ionogels (Fig. 3C). The DN ionogel with a crosslinking density of 0.5% exhibits a fracture energy of ~10,300 J/m^2^, which is the highest value reported to date for the direct ink writable ionogels (Fig. 3D and Fig. S6B). We attribute this high toughness to the existence of multiple dynamic crosslinking mechanisms between ZMPs and ILs (i.e., electronic interaction and hydrogen bonding), which provides DN ionogels with multiple efficient energy dissipating pathways during large deformation [14]. We also find that changing the size of the microparticles does not affect the mechanical behaviors of DN ionogels (Fig. S7). Furthermore, the intrinsic low melting point/glass transition temperature and nonvolatile properties of the ILs render the DN ionogel adaptable in a wide temperature range. Unlike the alginate-polyacrylamide hydrogel showing an ice-melting temperature around 0 °C, the DN ionogel exhibits a glass transition temperature at ~−84 °C, which is only slightly higher than the native ILs (*T*_g_ = −88 °C) (Fig. S8A). When placing the DN ionogel above the surface of the liquid nitrogen (~−50 °C), the DN ionogel can still be stretched over ~300% (Fig. S8B). The dynamic mechanical analysis (DMA) test further reveals that the DN ionogel can maintain its high elasticity even at −60 °C (Fig. 3E). The room temperature long-term stability of DN ionogel is examined. Before test, the DN ionogel is placed at 60 °C overnight and stored at room temperature to reach its equilibrium state. The nonvolatile feature of the IL ensures that the DN ionogels are capable of maintaining its weight and elasticity over several months (Fig. S9), while the alginate-polyacrylamide hydrogel lost its stretchability after 8 h, due to water evaporation. We further investigate the high-temperature stability of the DN ionogel. The thermogravimetric analysis (TGA) curve shows that the DN ionogel can maintain 92% of its initial weight at 220 °C, while the alginate-polyacrylamide hydrogel lost 94% of its weight at ~100 °C (Fig. S10). We also measure the stress–strain curves of the DN ionogels at high temperature ranging from 30 °C to 90 °C, and the results confirm that the stretchability of the ionogel is maintained at high temperature (Fig. 3F). Lastly, we monitor the change of Young’s moduli of the DN ionogels under different strain rates (Fig. 3G). The moduli of the DN ionogels increase from ~600 kPa at a small strain rate of <20 mm/min and saturate at ~2.75 MPa at a high strain rate of >40 mm/min (Fig. 3H).

**Fig. 4.**
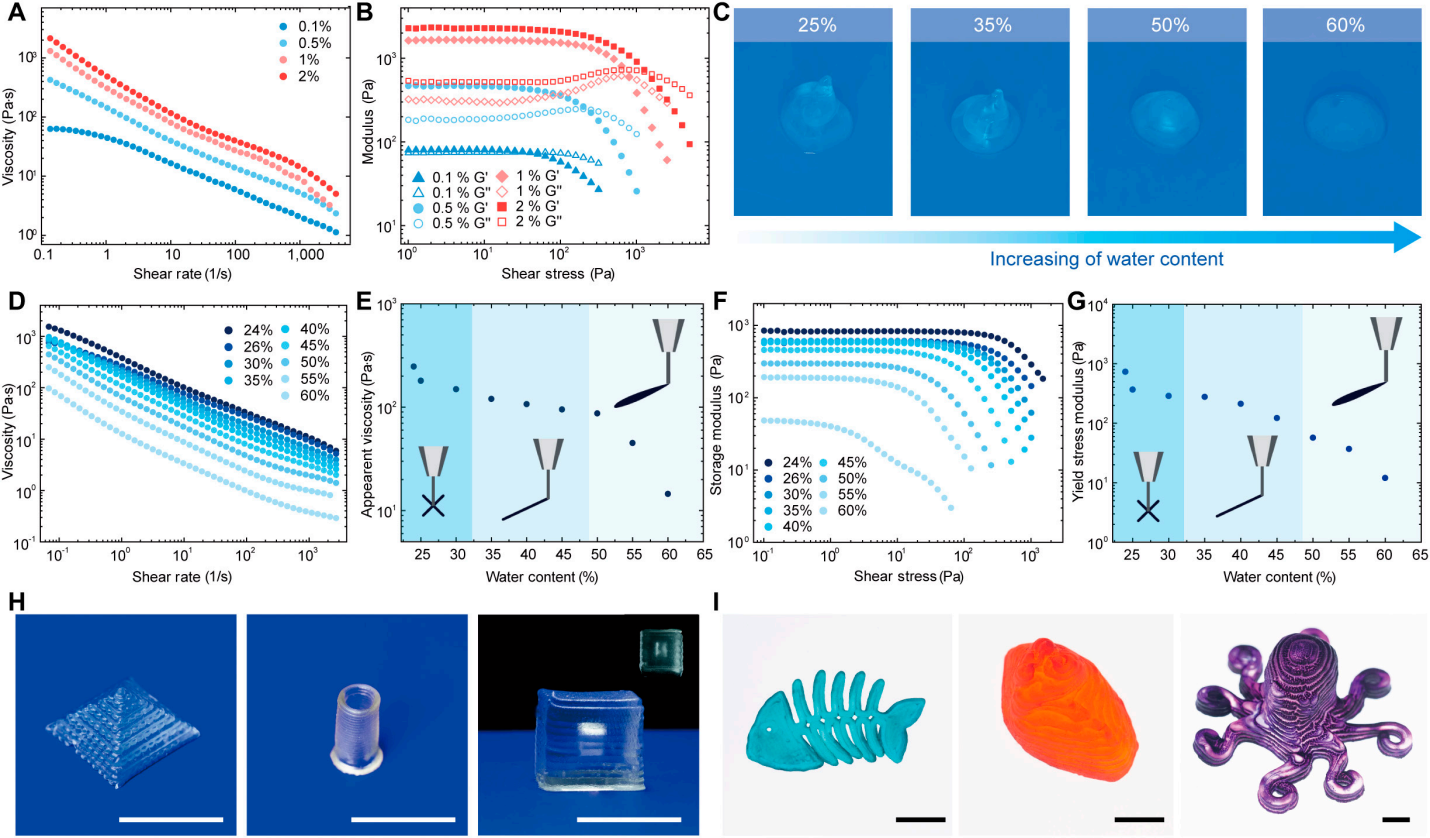
3D printing of double-network ionogel inks. (A) Viscosities of DN ionogel inks with different ZMP crosslinking densities (0.1%, 0.5%, 1%, and 2%) at different shear rates. (B) Storage and loss moduli of DN ionogel inks with different ZMP crosslinking densities (0.1%, 0.5%, 1%, and 2%) with varying shear stresses. (C) The DN ionogel ink transforms from semi-solid to thixotropic and finally to liquid state with increasing water content. (D) Apparent viscosity as a function of shear rate for DN ionogel inks with varying water content (24% to 60% by weight). (E) Apparent viscosity of DN ionogel inks as a function of water content (24% to 60% by weight). (F) Storage moduli as a function of shear stress for DN ionogel inks with varying water content (24% to 60% by weight). (G) Yield stress of DN ionogel inks as a function of water content (24% to 60% by weight). (H) A pyramid, a hollow cylinder, and a hollow cylinder-shaped 3D DN ionogel motifs are created using direct ink writable 3D printing (scale bar = 10 mm). (I) Fish bone-, semi-heart-, and octopus-shaped ionogels are 3D printed using dyed DN ionogel inks (scale bar = 10 mm).

The DN ionogel inks developed for direct ink writable must exhibit shear-thinning behavior to enable efficient flow through deposition nozzles without clogging, and rapidly recovering a solid-like response with a sufficiently high storage modulus and yield stress to maintain its shape upon printing. The DN ionogel inks are composed of ZMPs, ILs, water, AMPS monomer, MBAA, and *α*-ketoglutaric acid (photoinitiator). The rheological behaviors of microparticles are crucially dependent on their rigidity. To ensure that the ionogel inks possess sufficient storage modulus and yield stress, we first fine-tuned the crosslinking density of ZMPs from 0.1% to 2%. All inks exhibit shear-shinning behaviors without the need of addition of rheological modifiers (Fig. 4A). The oscillatory stress–strain measurements are carried out to determine the viscoelastic properties of the DN ionogel inks. The ionogel inks consisting of ZMP particles with high crosslinking density (0.5% to 2%) show predominantly elastic behavior at low shear rates (*G*′*>G*′) with a well-defined dynamic yield stress, which is favorable for direct ink writable (Fig. 4B). The printed ionogel structures with ionogel particle crosslinking density ≥0.5% retained their shapes, whereas the inks with particle crosslinking density below 0.5% form droplets (Fig. S11). To enable the efficient flow of ionogel inks, we compromise a portion of the intermolecular hydrogen bonding between the cation and anion molecules by adding water, which leads to weakened interaction between both the cationic and anionic molecules of ILs [45], lowering the apparent viscosities of the inks. As shown in Fig. 4C, with increasing water content from 24% to 60%, the DN ionogel inks are gradually transformed from semi-solid gels to 3D printable thixotropic gels and then to viscous gel liquids (Fig. 4C). The rheological behaviors of the DN ionogel inks with varied water content are determined to evaluate their printability (Fig. 4D to G and Fig. S12). The ideal printing window of the DN ionogel ink exists in the range between 31% and 45% water content. The DN ionogels that printed using ionogel inks with higher water content are less stretchable and softer (Fig. S13a). After a dehydration process (60 °C overnight), all the DN ionogels exhibit tough and highly stretchable mechanical behaviors, and their mechanical robustness in ambient condition is well maintained (Fig. S13b). The stability test is further investigated. As shown in Fig. S14, the DN ionogels printed using ionogel inks with different water content gradually dehydrate and reach their equilibrium state after ~48 h. The final water content in all DN ionogel samples is around 10%. A series of flower-shaped 2D patterns is 3D printed using different nozzle with diameters ranging from 90 to 600 mm (Fig. S15). The self-supportive nature of the DN ionogel ink enables the fabrication of 3D motifs like pyramid, hollow cylinders, and hollow cubes (Fig. 4H to J). We further printed more complex 2D and 3D architectures, demonstrating the excellent structural fidelity (Fig. S16).

**Fig. 5.**
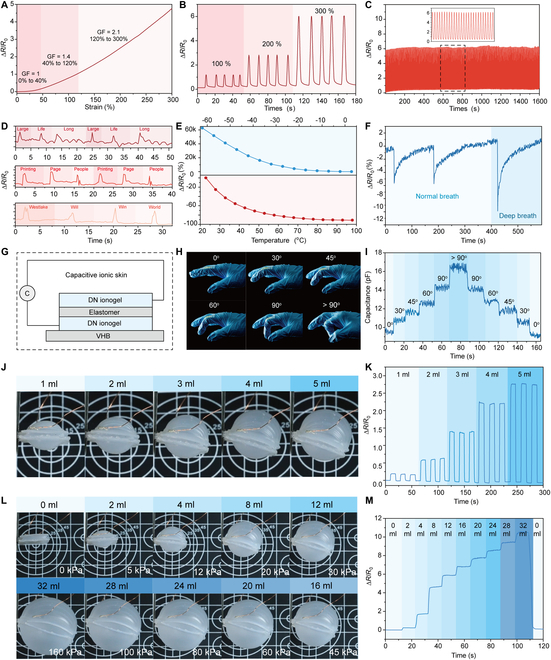
Fabrication and performance of ionogel-based ionotronics. (A) Relative resistance change and gauge factor of DN ionogel at varied strain (0% to 300%). (B) Relative resistance change of DN ionogel at fixed strains of 100%, 200%, and 300%. (C) The stability test of the DN ionogel under 300 stretching and relaxation cycles at 300% strain. (D) Sensing of muscular motion of the throat when saying “Large, Long, Life”, “Printing, Page, People”, and “Westlake, Will, Win, World”. (E) Relative resistance change of DN ionogel at low (top) and high temperature (bottom). (F) The DN ionogel functions as a humidity sensor sensing normal and deep breath. (G) Illustration of DN ionogel-based ionic skin design. (H and I) Determination of capacitance change of ionogel-based ionic skin at different bending angles. Relative resistance change of ionogel sensor-equipped pneumatic soft actuators at (J and K) small and (L and M) large deformation.

The tunable mechanical property and ionic conductivity make the DN ionogels an excellent candidate for sensors. When stretched, the ionogel resistance increases with strain (Fig. 5A). The gauge factors in the strain range of 0% to 40%, 40% to 120%, and 120% to 300% are calculated as 1, 1.4, and 2.1, respectively. The strain-dependent resistance change is also highly stable and repeatable (Fig. 5B). We then record the continuous resistance change of DN ionogel at fixed strains of 100%, 200%, and 300% for 300 cycles, demonstrating its long-term durability (Fig. 5C and Fig. S17). Further adhering DN ionogel to the surface of throat, the resistance changes of DN ionogel can be used to detect pronunciation (Fig. 5D). Since the ion mobility is governed by temperature, the DN ionogel is also temperature sensitive; it exhibits extremely high sensitivity at low temperature and moderate sensitivity at high temperature (Fig. 5E). In addition, the DN ionogel can sense humidity changes during breathing. When breathing toward the ionogel, the resistance decreases immediately and recovers to its initial value after ~100 s (Fig. 5F). The deep breath can induce a larger decrease in resistance. We then produce an ionogel skin by printing DN ionogels and silicone elastomer on VHB substrate as illustrated in Fig. 5G. The sandwich-like sensor consists of a top and bottom layer of DN ionogels as the electrodes, and a middle layer of silicone elastomer as the dielectric layer. When adhering the sensor on top of a human hand, the ionogel skin can function as a motion sensor detecting different bending angles of the finger via recording the capacitance signal changes between the 2 electrodes (Fig. 5H and I).

The mechanical softness and large-strain sensing ability of the DN ionogel makes it a promising sensing unit for soft robots. Here, we bond DN ionogel with silicone-based elastomers via a modified silane coupling strategy [33], leading to a DN ionogel sensor integrated pneumatic soft actuator. We first formulate both the 3D printable DN ionogel ink and elastomer ink with the addition of a small amount of water-soluble silane coupling reagent 3-(trimethoxysilyl)propyl methacrylate (TMSPMA). The strong covalent linkages between DN ionogel and silicone elastomer can be formed via silane condensation reaction at elevated temperatures. The SEM image shows that the DN ionogel forms an intimate interface with PDMS (Fig. S18). The adhesion energy between the DN ionogel and elastomer is determined as ~250 J/m^2^ (Fig. S19), which prevents the delamination of the DN ionogel sensors during large deformation (~800%) and improves the sensing stability of DN ionogel sensors (Fig. S20). Next, we print a serpentine-shaped DN ionogel strain sensor on top of a pneumatic soft actuator. After curing, the performance of ionogel sensor-equipped soft actuator is then tested. Under small deformation, the ionogel sensor exhibits highly repeatable and stable resistance change during inflation–deflation cycles (Fig. 5J and K and Movie S1). We further monitor the resistance change of the ionogel sensor under larger deformations. The pneumatic soft actuator is inflated stepwise from 0 to 160 kPa and then deflated, the resistance of ionogel increases with input pressures simultaneously and recovers rapidly (less than 5 s) at rest (Fig. 5L and M and Movie S2). During the actuation cycles, the DN ionogel sensor remains adhered to the surface of actuators, and no delamination is observed even after 50 cycles.

**Fig. 6.**
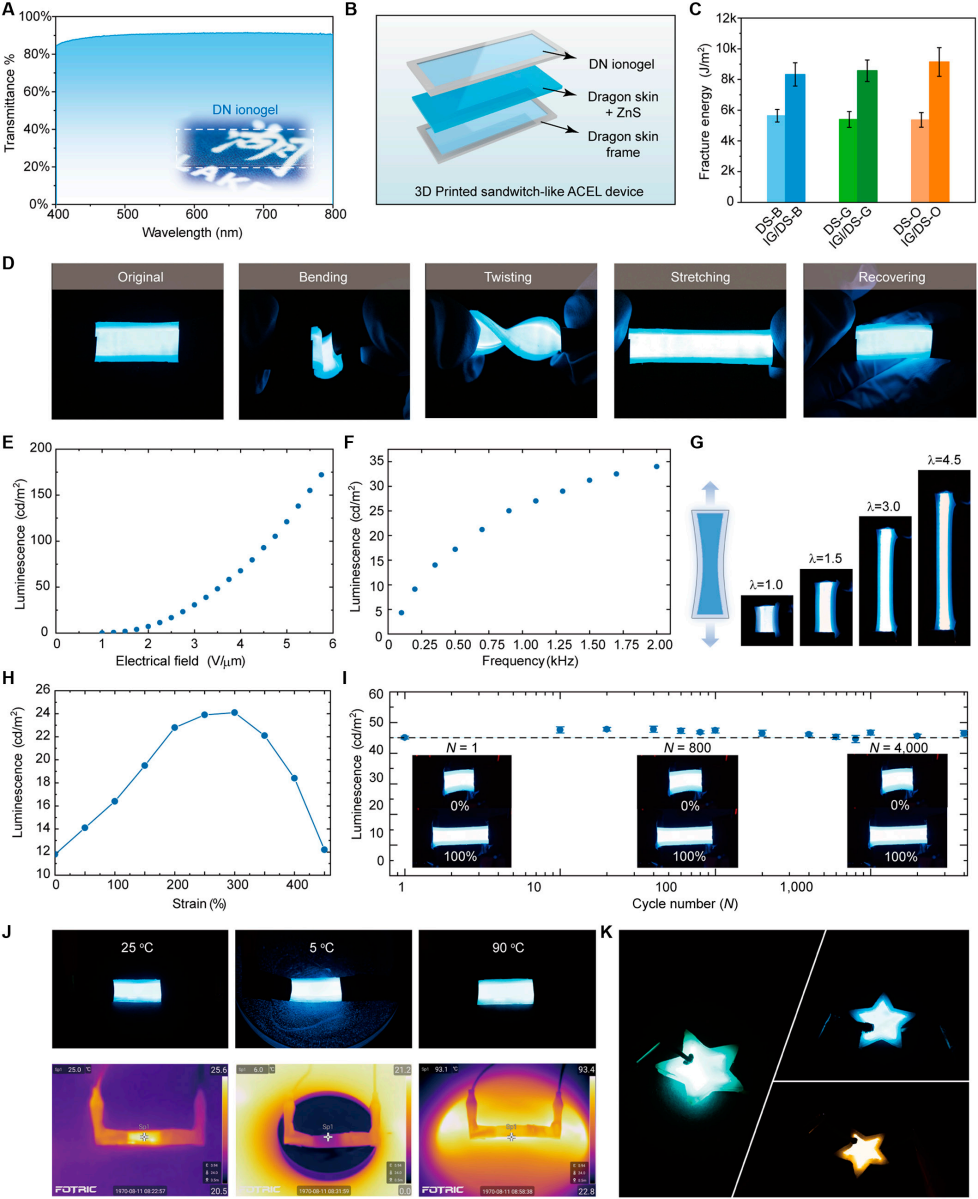
Performance of ionogel-based ACEL devices. (A) Optical transmittance spectrum of DN ionogel in the visible range. (B) Schematic illustration of ACEL device design. (C) Investigation of fracture energy of ACEL devices and elastomer/ZnS. (D) Photographs of the light-emitting performance of the printed ACEL devices under various deformations: bending, twisting, and stretching. (E) Luminance as a function of electric field. (F) Luminance as a function of frequency with an electric field of 2.5 V/μm. (G) Photographs of light-emitting performance of the ACEL device under large deformation at a fixed electric field of 2.5 V/μm and a fixed frequency of 2 kHz. (H) Change of luminance as a function of strains in the range from 0% to 450%. (I) Cyclic stretching test of the printed ACEL device stretched to 200% of its original length (*n* = 3). Inset: Images of the ACEL device after 1, 800, and 4,000 stretching cycles. (J) The digital photographs (top) and IR spectrometer photographs (bottom) of the ACEL device at different temperatures. (K) Demonstrations of printed ACEL devices with arbitrary shapes.

For our last demonstration, we harness the high optical transparency and ionic conductivity of the DN ionogel, as well as its mechanical robustness, for the fabrication of ionogel-based ACEL devices with both high stretchability and toughness. The UV-Vis transmittance spectra of the DN ionogel with a thickness of 1 mm show an average transmittance of >90% in the visible spectral range, which is favorable for fabrication of transparent electrodes (Fig. 6A). We then synthesize the luminescence layer ink by mixing the ZnS particles with PDMS and silane coupling agent. Via multimaterial 3D printing layer-by-layer, a sandwich-like ACEL device with blue emission is manufactured (Fig. 6B). The high toughness of the DN ionogel and strong covalent linkages between the electrode and luminescent layer further improve the mechanical properties of the printed ACEL device (Fig. 6C); i.e., the printed device well maintains its stable light emission under various deformation, such as bending, twisting, and stretching (Fig. 6D). Next, we measure the dependence of luminescence on the applied electric fields. At a frequency of 2 kHz, the blue light emissive ACEL device exhibits a threshold electric field of ~ 1V/μm, above which its luminescence increases with increasing of applied electric field due to the increased number of charge carrier recombination and excited luminescence centers (Fig. 6E). The maximum luminescence of the printed ACEL device reaches up to ~170 cd/m^2^ with an applied electric field of ~5.6 V/μm. Subject to a fixed electric field of ~2.5 V/μm, the luminescence of printed ACEL device increases with increasing applied AC frequency (Fig. 6F), fitting well with the relation of *I* = *aexp*(−*b*/*V*^1/2^), where *I* is the luminescence intensity, and *a* and *b* are the fitting parameters. In addition, the emission color of the ACEL device blue shifts with increasing applied frequency (Fig. S21). The emission performance of ACEL device under large deformation is further tested. The strong covalent bonding between DN ionogel electrodes and the emission layer renders the device stable, exhibiting uniform light emission under a maximum strain of ~ 450% (Fig. 6G). Further strain increase results in delamination between the DN ionogel electrode and the conductive copper plate, resulting in device failure. We then characterize the light emission as a function of strain at a fixed electric field of ~2 V/μm and a frequency of 2 kHz (Fig. 6H). The luminescence intensity of the ACEL device increases with increasing applied strain, and it reaches maximum of ~24 cd/m^2^ at ~300% strain, with a further increase of the applied strain lowering the luminescence intensity. We also examine the durability of the printed ACEL device under cyclic stretching test at a strain of 100% up to 4,000 cycles, showing minimal degradation of luminescence intensity (Fig. 6H and Movie S3). In addition, the printed ACEL device also delivers stable performance within a wide temperature range from ~5 to 95 °C, owing to the thermal stability of DN ionogels (Fig. 6J). Finally, a series of 2.5D shaped ACEL devices with different emission colors and robust mechanical properties are printed, demonstrating the high versatility and manufacturability of our DN ionogel ink (Fig. 6K).

## Discussion

In summary, we developed a novel and general strategy to formulate stable and printable DN ionogel inks by employing granular polymeric microparticles to stabilize ILs that function as the physical crosslinkers, providing ionogels with efficient energy dissipation networks. The DN ionogel ink possesses desirable rheological properties, which enable the fabrication of 3D DN ionogel motifs as well as ionotronic sensors. Via covalently adhering DN ionogels to PDMS, we integrated DN ionogels onto pneumatic soft actuators and demonstrate their capabilities in sensing large deformation. We further harness multimaterial 3D printing to manufacture a series of tough and stretchable ACEL devices with tunable light emission properties. We envision that by choosing different polymeric particles with high IL affinity, our approach can also be extended to other polymer/IL systems. Overall, our printable microparticle-based ionogel ink represents a versatile platform for the future manufacturing of ionotronics.

## Materials and Methods

### Materials

The branched PEI (weight average molecular weight of 25 kDa, Sigma-Aldrich), AAc (Sigma-Aldrich), AMPS (TCL), MBAA (Sigma-Aldrich), [EMIM][EtSO_4_] (TCL), Irgacure 2959 (Sigma-Aldrich), α-ketoglutaric acid (*α*-KA, Sigma-Aldrich), hydrogen peroxide (H_2_O_2_, Sigma-Aldrich), Dragonskin (DS, Smooth-on, 10), polyoxyethylene lauryl ether (Brij 35, Sigma-Aldrich), TMSPMA (TCL), and ZnS phosphor powder (D512C Green, D502B Blue, and D611S Orange, Shanghai Keyan Phosphor Technology Co., Ltd., China) are used as received. The deionized Milli-Q water is used as dispersion medium.

### Synthesis of PEI-*co*-PAA zwitterionic microparticles

PEI (2.5 g) is first dissolved in 40 ml of DI water, followed by the addition of appropriate amount of AAc (8.3, 12.5, and 25 g) and MBAA (0.1, 0.5, 1, and 2 wt% of monomer). The mixture is degassed by purging N_2_ for 30 min. Then, 1 ml of 100 mM H_2_O_2_ is added into the mixture. The mixture is heated up to 80 °C to initiate the copolymerization process. At 80 °C, H_2_O_2_ can generate free radicals on the amine groups, which initiate the graft copolymerization of AAc monomers. In addition, the H_2_O_2_ can also generate hydroxyl radicals, which can initiate the homopolymerization of AAc monomers. The mixtures gradually transform from clear solution to hydrogel after 1 h (if the gelation does not happen in the first hour, additional H_2_O_2_ or ammonium persulfate can be added to accelerate the gelation process). To improve the monomer conversion, the reaction is allowed to react for 4 h (in the first hour, the monomer conversion is 85 to 90%). Thereafter, the zwitterionic hydrogel is immersed into the DI water and dialyzed to neutral pH for at least 1 week. The neutralized zwitterionic hydrogel is further freeze-dried for 5 days to form dried zwitterionic copolymers. The copolymer is then ground by ball-milling for 4 days forming zwitterionic microparticles. The zwitterionic microparticles are filtered through 400-μm, 200-μm, 100-μm, and 50-μm filters to produce microparticles with desired particle sizes.

### Preparation of DN ionogel ink

The 0.4 g of zwitterionic microparticles and 2.4 g of [EMIM][EtSO_4_] and 1.8 g of DI water are mixed with a planetary mixer (Thinky ARE-300) at 2,000 revolutions per minute (RPM) for 2 min and then stored for 1 day to guarantee the full swelling of zwitterionic microparticles. The mixtures are then filtered by a 10-micron filter to remove large aggregates. Thereafter, 2.4 g of AMPS, 0.012 g of MBAA, and 0.07 g of α-KA are added into the mixtures and mixed with a planetary mixer at 2,000 RPM for 2 min.

### Preparation of modified DN ionogel ink

The DN ionogel ink is prepared by the above-mentioned recipe, followed by the addition of 10.72 μl of TMSPMA and 56 μl of acetic acid (0.1 M). The mixture is then mixed with a planetary mixer at 2,000 RPM for 2 min, fabricating the modified DN ionogel ink. The modified DN ionogel ink is loaded into a syringe and centrifuged at 3,000 RPM for 5 min before printing.

### Preparation of the modified elastomer ink

The 0.08 g of Brij 35 is added into 2 g of DS part A, and the mixture is heated to 60 °C to melt the surfactant. Thereafter, the mixture is mixed with a planetary mixer at 2,000 RPM for 1 min, followed by the addition of 0.08 ml of TMSPMA, 0.08 g of retarder, and 2 g of DS part B. The mixture is again mixed using a planetary mixer at 2,000 RPM for 1 min. The modified elastomer ionogel ink is then loaded into a syringe and centrifuged at 3,000 RPM for 5 min before print.

### Preparation of the modified elastomer/ZnS ink

The 0.04 g of Brij 35 is added into 1 g of DS part A, and the mixture is heated to 60 °C to melt the surfactant. Thereafter, the mixture is mixed with a planetary mixer at 2,000 RPM for 1 min, followed by the addition of 0.04 ml of TMSPMA, 0.04 g of retarder, and 2 g of ZnS phosphor. The mixture is then mixed using a planetary mixer at 2,200 RPM for 3 min. Thereafter, 1 g of DS B is added into the mixture, and again mixed at 2,200 RPM for 3 min. The elastomer/ZnS ink is then loaded into a syringe and centrifuged at 3,500 RPM for 10 min before print.

### 3D printing of DN ionogels and DN ionogel-based sensors

Direct ink writable is carried out using a 4-axis custom-made 3D printer. The printable ink (DN ionogel ink or elastomer ink or elastomer/ZnS ink) is housed in a syringe (3 ml, EFD Inc., East Province, RI, USA) attached by Luer-lock to a commercial nozzle (90, 150, 330, 450, or 600 mm, Nordson EFD). The printing path is generated via the production of G-code that outputs the XYZ motion of the custom-made 3D printer. G-code is generated either by hand or by using Regenovo software. The DN ionogels and ionogel-based sensors are printed on PMMA substrate and UV-cured for 2 h (Hamamatsu LC8, LIGHTNINGCURETM) and then thermally treated at 60 °C overnight. The cured samples were peeled off from the PMMA substrate and stored in a petri dish at room temperature (20 to 25 °C, ~50% relative humidity). The final water content of the DN ionogel or DN ionogel-based sensors is ~10%.

### 3D printing of DN ionogel integrated pneumatic soft actuators

The pneumatic soft actuator is 3D printed in the carbomer supporting bath according to our previous report [46]. The 0.3% w/w carbomer is first dissolved in DI, followed by the addition of a small amount of 10 M NaOH forming a uniform supporting bath. The as-prepared supporting bath is further degassed in vacuum and ready for printing. The elastomer ink is composed of 2 g of DS part A, 2 g of DS B, 0.04 g of Brij 35, 0.04 ml of TMSPMA, and 0.04 g of retarder. The pneumatic soft actuator is 3D printed using a 4-axis custom-made 3D printer inside the supporting bath and then cured at 80 °C overnight. The cured soft actuator is then taken out of the gel matrix and washed 3 times to remove the remaining carbomers. The serpentine-shape DN ionogel sensor is then deposited on top of the actuator by direct ink writable printing. After printing, the DN ionogel is cured by a UV light for 10 min. The DN ionogel integrated pneumatic soft actuator is further placed in the oven (70 °C) for 24 h and stored at room temperature before use.

### 3D printing of ACEL devices

The ACEL device is printed using the multimaterial 3D printing technique. For printing a planar ACEL device, an elastomer frame is first printed via using DS ink with silane coupling reagent. Then, the DN ionogel ink is deposited inside the elastomer frame via 3D printing serving as a bottom electrode. After in situ UV curing of ionogel for 10 min, the elastomer/ZnS layer is printed on top of the DN ionogel. Thereafter, another elastomer frame is printed on top of the elastomer/ZnS layer, followed by printing DN ionogel inside the elastomer frame. After UV curing of the DN ionogel. The device is placed in an oven at 60 °C for 24 h and stored at room temperature before use.

### Rheological characterization

All the rheological measurements are determined with a controlled stress rheometer (DHR-3, TA Instruments, New Castle, DE), using a 25-mm-diameter plane plate with a 500-μm gap between the parallel plates at 25 °C. The viscosities of samples are measured with a steady shear rate increasing from 0.01 to 1,000 s^−1^, at a frequency of 1 Hz. The storage and loss modulus measurements are performed in an oscillatory mode, in which the stress amplitude ranged from 0.1 to 10,000 Pa at a stress sweep with a frequency of 1 Hz.

### Mechanical testing

The DN ionogel ink is 3D printed into tensile specimens and cured under UV irradiation for 10 min. The samples are then placed on a heating plate at 60 °C overnight, and left at room temperature for 1 day. The samples are tested on a mechanical testing machine (Instron) with a 5-N load cell at a rate of 10 mm/min.

### Mechanical test of ionogel under low and high temperatures

The mechanical properties of DN ionogels under low (−60 to 0 °C) and high (30 to 90 °C) temperatures are measured by a TA Discovery DMA 850 dynamic mechanical analyzer (DMA). The DN ionogel samples are fabricated with a size of 20 × 5 × 1 mm (length × width × thickness). Before testing, the samples are placed in the DMA test chamber, allowing samples to reach the targeted temperatures. All the samples are tested at a rate of 10 mm/min.

### 90° peeling test

The modified DS ink is first printed onto a plasma-treated glass substrate. Thereafter, the DN ionogel is 3D printed on top of DS surfaces following the above-mentioned procedure with a size of 60 × 20 × 2 mm (length × width × thickness). The printed samples are heated at 60 °C overnight and left at room temperature for 1 day. As a stiff backing layer for the ionogel, PETE film is bonded onto the ionogel via cyanoacrylate adhesive, preventing the elongation of ionogel along the peeling direction. The resultant samples were tested with the standard 90° peeling test with a constant peeling speed of 60 mm min^−1^. The measured peeling force reached a plateau (with slight oscillations), as the peeling process entered steady state.

### Differential scanning calorimetry characterization

The glass transition temperature of the DN ionogel and the freezing temperature of alginate-polyacrylamide hydrogel are determined using Perkin-Elmer DSC 7 via scanning a temperature range from −100 °C to 40 °C (10 °C/min) under flowing N_2_.

### TGA measurements

The TGA measurement is performed on a Mettler-Toledo TGA/DSC 3+/1600 HT via scanning a temperature range from 70 to 800 °C (10 °C/min) under flowing air.

### Scanning electron microscopy

The morphologies of zwitterionic microparticles are characterized using a scanning electron microscope (SEM) (Gemini450, Zeiss, Germany). The ground zwitterionic microparticles are first filtered with a 100-mm stainless steel mesh and then deposited on a conductive tape. The sample is coated with a 5-nm gold film before characterization.

### Ionic conductivity measurement

The SN ionogel is first deposited into a custom-made mold (L = 10 mm, W = 2 mm, H = 0.1 mm) and then transferred to a glass substrate with 2 electrodes distanced at 8 mm. The ionic conductivity of SN ionogels is measured using an LCR meter (E4980AL, Keysight, USA), the applied voltage is 1 V, and the measuring frequency is 1 kHz. The ionic conductivity (*s*) is calculated by

s=L/RA (1)

where *R* is the resistance, and *L* and *A* are the length and cross-sectional area, respectively.

### Resistance and capacitance measurement of ionogel-based ionotronic sensors

The resistance of the resistive DN ionogel sensor and the capacitance of the DN ionogel skin is measured using an LCR meter (E4980AL, Keysight, USA), respectively. To measure gauge factor, resistance change at fixed strain, and cycling performance of the DN ionogel, the sample is mounted on a home-built desktop stretcher with 3D printed clamps. The copper clamps are used to connect the DN ionogel with an LCR meter. The resistance change of the DN ionogel during stretching is recorded in real time. To investigate the capacitance change of the DN ionogel skin during bending, the DN ionogel skin is attached to a plastic glove, and 2 copper wires are inserted into both top and bottom ionogel electrodes, and connected to an LCR meter. The capacitance change of DN ionogel skin is recorded by an LCR meter during bending.

### Resistance measurement of DN ionogel integrated pneumatic soft actuator

To measure the resistance change of the DN ionogel integrated pneumatic soft actuator during its large deformation, 2 copper wires are inserted into the ionogel sensor and connected to the LCR meter. The resistance change of the DN ionogel is recorded in real time during expansion of the soft actuator.

### Transparent test

The transparency of the DN ionogel is characterized using a UV-Vis spectrophotometer (Shimadzu 2700). The wavelength for the test is set from 400 to 800 nm.

### ACEL performance characterization

The luminance and emission spectra of ACEL devices are recorded using a luminance meter (OHSP-350Z, HongPu Optoelectronics Technology Co., Ltd., China). The ACEL device is powered by a high-voltage AC/DC generator (Model 615-3, Trek, USA). For cycling test, the ACEL device is mounted on a home-built desktop stretcher with 3D printed clamps. The top and bottom DN ionogel electrodes are connected with conducting copper tape. Two copper clamps are used to connect the AC/DC generator and conducting copper tape. The cycling test is performed at a speed of 1 mm/s. The luminance of the ACEL device is in situ monitored by a luminance meter.

## Data Availability

All data needed to support the conclusions in the paper are provided in the paper and the Supplementary Materials.

## Supplementary Materials

Supplementary 1 Fig. S1. The FTIR spectrum of the PEI-*co*-PAA zwitterionic copolymer. Fig. S2. (A) The pH-dependent swelling behavior of the PEI-*co*-PAA zwitterionic copolymer. Fig. S3. Fabrication of zwitterionic microparticles. Fig. S4. SEM images of ball-milled zwitterionic microparticles. Fig. S5. The squeeze test of SN ionogels with varied ZMP-to-IL weight ratios under (A) dry and (B) humid conditions. Fig. S6. (A) The force–distance of DN ionogels with a varying ZMPs/AMPS molar ratio (1:3, 1:4, 1:5, 1:6, and 1:7). Fig. S7. (A) The optical image of zwitterionic microparticles with an average diameter of ~10 μm. Fig. S8. (A) The differential scanning calorimetry curve of native ionic liquid, the PAAm-alginate DN hydrogel, and the DN ionogel. Fig. S9. Stability test of the DN ionogel and the alginate-polyacrylamide hydrogel for 3 months. Fig. S10. The TGA curves of the DN ionogel and the DN hydrogel. Fig. S11. The ionogel inks consisting of ZMPs with varied crosslinking density are printed on the substrate. Fig. S12. Shear storage and loss modulus as a function of shear force for DN ionogel inks with different water content. Fig. S13. (A) The strain–stress curves of DN ionogel specimens printed via DN ionogel inks with different water content (35%, 40%, and 45%). Fig. S14. The stability test of DN ionogel specimens printed via DN ionogel inks with different water content (35%, 40%, and 45%). Fig. S15. Printing follower-shaped ionogels via nozzles with different diameters. Fig. S16. Printed DN ionogel octopus under compression (top) and stretching (bottom). Fig. S17. The stability test of DN ionogel under 300 stretching and relaxation cycles at 100% and 200% strain. Fig. S18. (A) The SEM image of the morphology of the interface between the DN ionogel and PDMS (scale bar = 10 μm). Fig. S19. (A) Curves of the peeling force per width of ionogel sheet versus displacement for ionogel–elastomer bonding. Fig. S20. (A) The DN ionogel-based capacitive ionic skin with covalent linkage between DN ionogels and PDMS in sensing bending–unbending of fingers. Fig. S21. (A) The electroluminescence spectra of the ACEL device with varying applied frequencies (100, 500, 1,000, 2,500, and 5,000 Hz).

Supplementary 2 Movie S1. Small deformation cyclic test of the ionogel sensor-equipped pneumatic soft actuator. Movie S2. Large deformation test of the ionogel sensor-equipped pneumatic soft actuator. Movie S3. 3D printed ACEL device under stretching cycles (100 out of 4,000 cycles).
