# Supplementary material for: Granular Ionogel Particle Inks for 3D Printed Tough and Stretchable Ionotronics
Source: Research (Wash D C). 2023 Jun 7;6:0104. doi: 10.34133/research.0104 (PMC10246561; doi:10.34133/research.0104)
Supplement: Supplementary 1 — Fig. S1. The FTIR spectrum of the PEI-co-PAA zwitterionic copolymer. Fig. S2. (A) The pH-dependent swelling behavior of the PEI-co-PAA zwitterionic copolymer. Fig. S3. Fabrication of zwitterionic microparticles. Fig. S4. SEM images of ball-milled zwitterionic microparticles. Fig. S5. The squeeze test of SN ionogels with varied ZMP-to-IL weight ratios under (A) dry and (B) humid conditions. Fig. S6. (A) The force–distance of DN ionogels with a varying ZMPs/AMPS molar ratio (1:3, 1:4, 1:5, 1:6, and 1:7). Fig. S7. (A) The optical image of zwitterionic microparticles with an average diameter of ~10 μm. Fig. S8. (A) The differential scanning calorimetry curve of native ionic liquid, the PAAm-alginate DN hydrogel, and the DN ionogel. Fig. S9. Stability test of the DN ionogel and the alginate-polyacrylamide hydrogel for 3 months. Fig. S10. The TGA curves of the DN ionogel and the DN hydrogel. Fig. S11. The ionogel inks consisting of ZMPs with varied crosslinking density are printed on the substrate. Fig. S12. Shear storage and loss modulus as a function of shear force for DN ionogel inks with different water content. Fig. S13. (A) The strain–stress curves of DN ionogel specimens printed via DN ionogel inks with different water content (35%, 40%, and 45%). Fig. S14. The stability test of DN ionogel specimens printed via DN ionogel inks with different water content (35%, 40%, and 45%). Fig. S15. Printing follower-shaped ionogels via nozzles with different diameters. Fig. S16. Printed DN ionogel octopus under compression (top) and stretching (bottom). Fig. S17. The stability test of DN ionogel under 300 stretching and relaxation cycles at 100% and 200% strain. Fig. S18. (A) The SEM image of the morphology of the interface between the DN ionogel and PDMS (scale bar = 10 μm). Fig. S19. (A) Curves of the peeling force per width of ionogel sheet versus displacement for ionogel–elastomer bonding. Fig. S20. (A) The DN ionogel-based capacitive ionic skin with covalent linkage betwee [file research.0104.f1.docx]

**Granular Ionogel Particle Inks for 3D Printed Tough and Stretchable Ionotronics**

Yuan Yao^1,2^, Yue Hui^1,2,3^, Zhenhua Wang^1,2^, Hehao Chen^1,2^, Heng Zhu^4^, Nanjia Zhou^1,2^*

^1^Key Laboratory of 3D Micro/Nano Fabrication and Characterization of Zhejiang Province, School of Engineering, Westlake University, Hangzhou, 310024, Zhejiang Province, China.

^2^Institute of Advanced Technology, Westlake Institute for Advanced Study, Hangzhou 310024, Zhejiang Province, China.

^3^School of Chemical Engineering and Advanced Materials, the University of Adelaide, Adelaide, 5005, South Australia, Australia.

^4^State Key Laboratory of Fluid Power & Mechatronic System, Key Laboratory of Soft Machines and Smart Devices of Zhejiang Province, Center for X-Mechanics, Department of Engineering Mechanics, Zhejiang University, Hangzhou 310027, China

^*^Address correspondence to: Nanjia Zhou; [zhounanjia@westlake.edu.cn](mailto:zhounanjia@westlake.edu.cn)


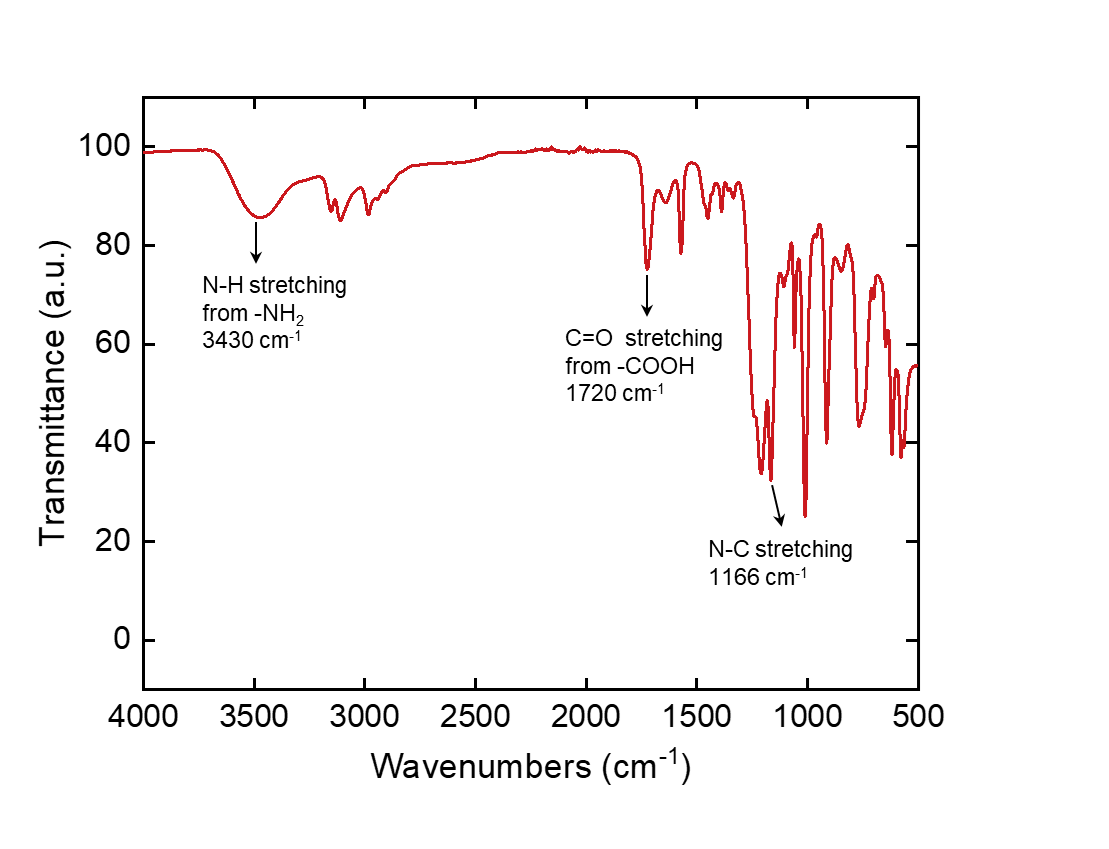


**Fig. S1.** The FTIR spectrum of PEI-*co*-PAA zwitterionic copolymer.


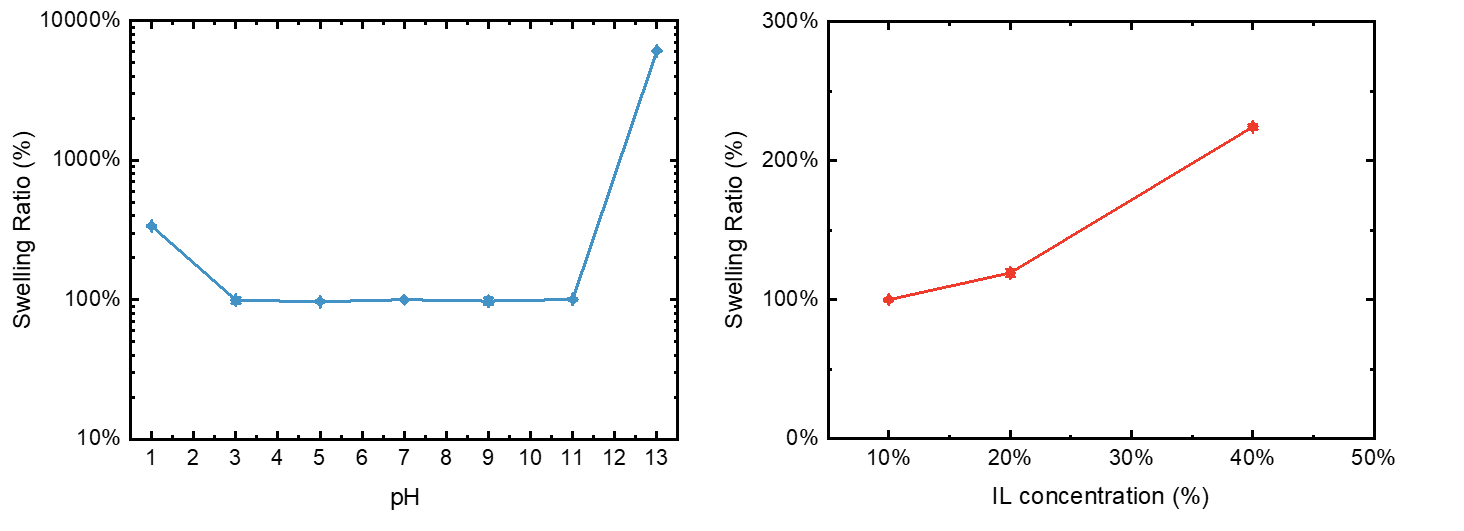


**Fig. S2.** (a) The pH dependent swelling behavior of PEI-*co*-PAA zwitterionic copolymer; (b) The “anti-polyelectrolyte effect” of PEI-*co*-PAA zwitterionic copolymer in ionic liquid/water mixture solutions with different ionic liquid concentrations.


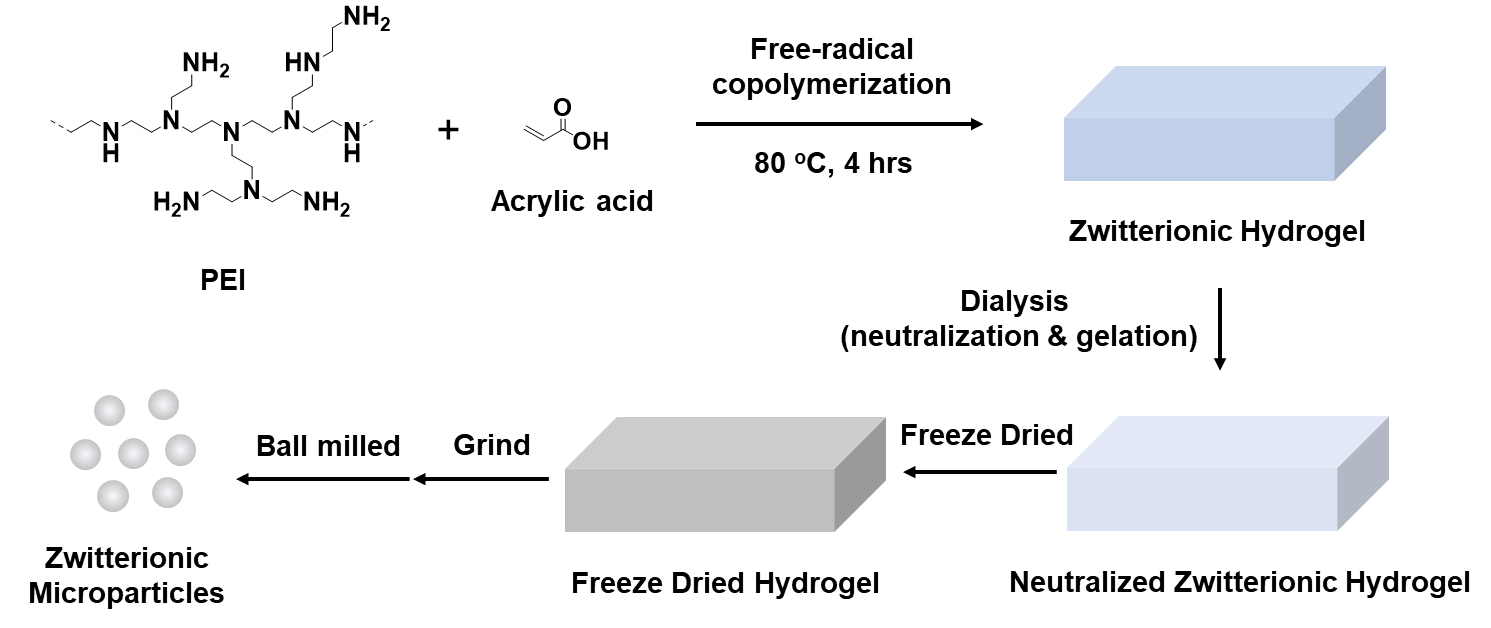


**Fig. S3.** Fabrication of zwitterionic microparticles.


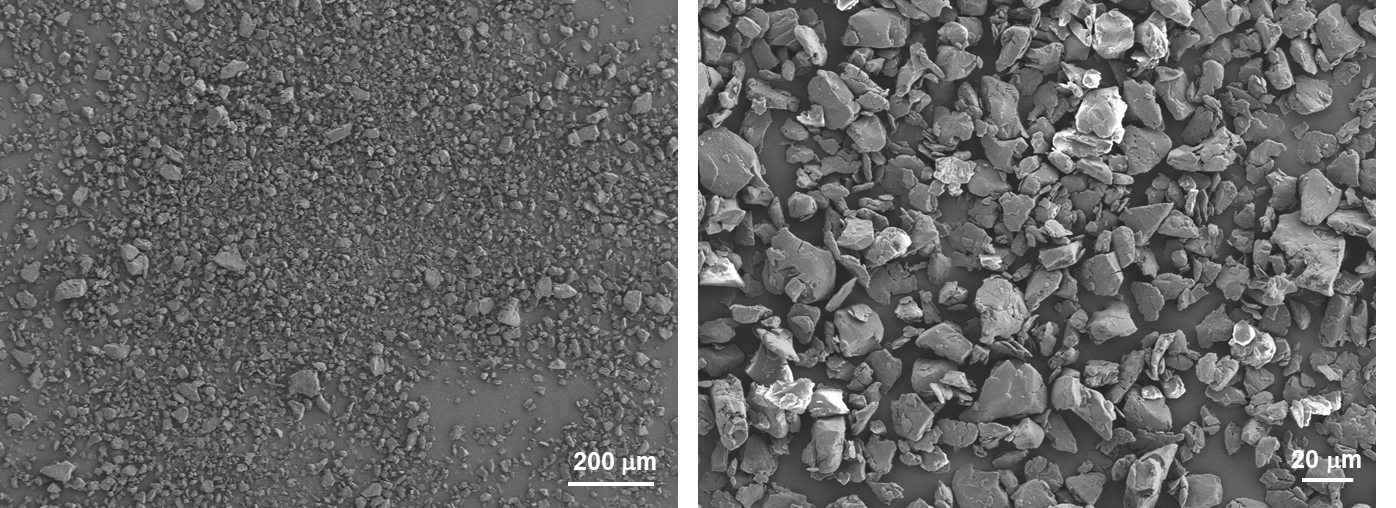


**Fig. S4.** SEM images of ball-milled zwitterionic microparticles.


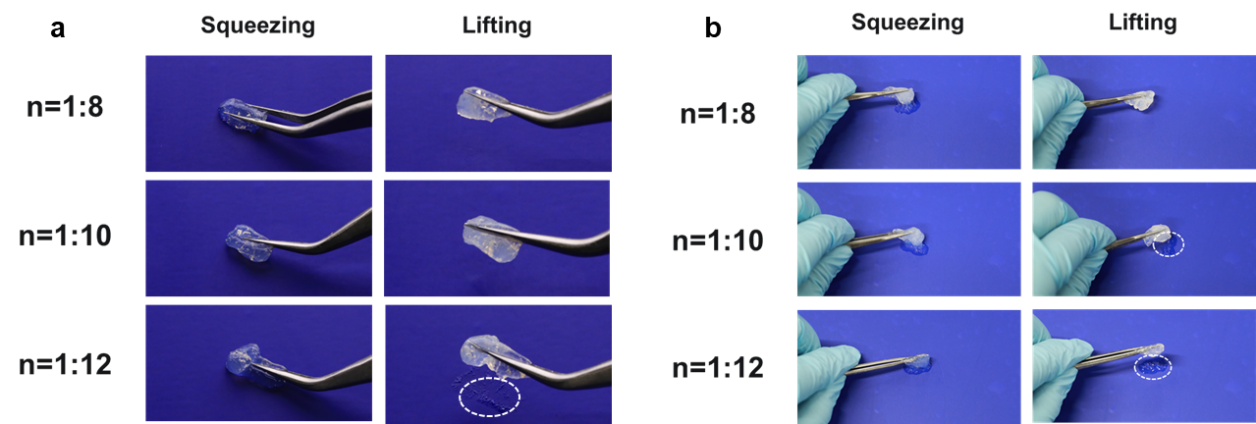


**Fig. S5.** The squeeze test of SN-ionogels with varied ZMP to ILs weight ratios at (a) dry and (b) humid condition.


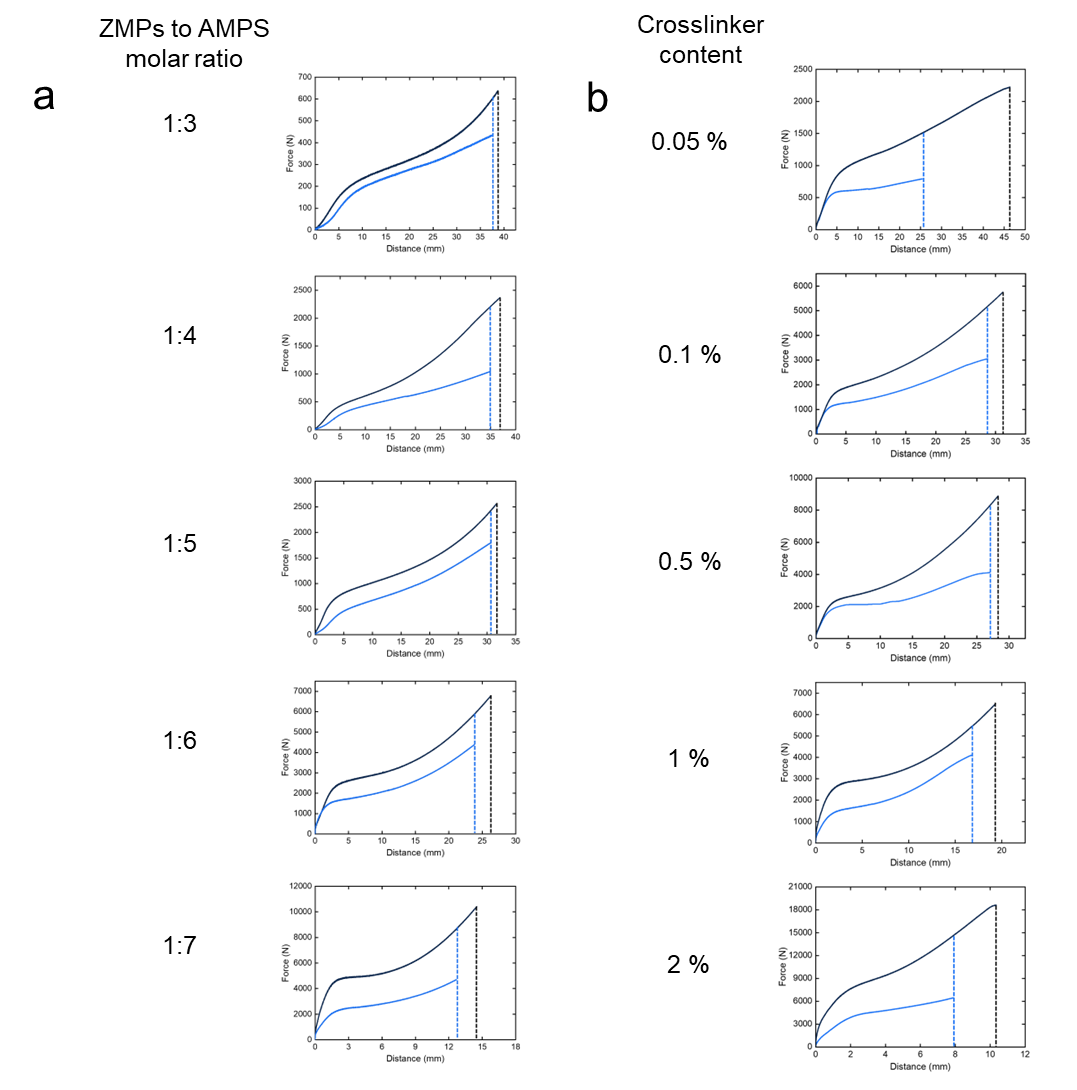


**Fig. S6.** (a) The force-distance of DN ionogels with a varying ZMPs/AMPS molar ratio (1:3, 1:4, 1:5, 1:6 and 1:7). (b) The force-distance of DN ionogels with a varying crosslinking density (0.05%, 0.1%, 0.5%, 1% and 2%) and a fixed ZMPs to AMPS molar ratio of 1: 6. Black and blue line indicate the unnotched and notched ionogel samples, respectively.


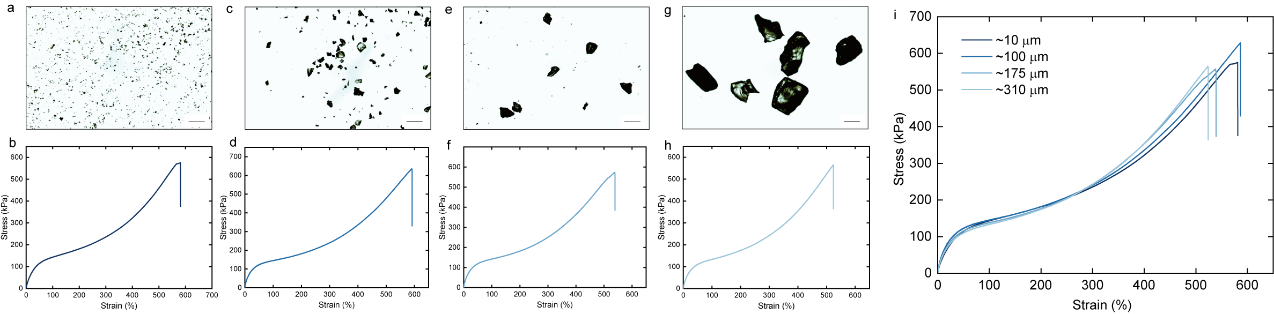


**Fig. S7.** (a) The optical image of zwitterionic microparticles with an average diameter of ~ 10 μm; (b) The strain-stress curve of DN ionogels fabricated using zwitterionic microparticles with an average diameter of ~ 10 μm; (c) The optical image of zwitterionic microparticles with an average diameter of ~ 100 μm; (d) The strain-stress curve of DN ionogels fabricated using zwitterionic microparticles with an average diameter of ~ 100 μm; (e) The optical image of zwitterionic microparticles with an average diameter of ~ 175 μm; (f) The strain-stress curve of DN ionogels fabricated using zwitterionic microparticles with an average diameter of ~ 175 μm; (g) The optical image of zwitterionic microparticles with an average diameter of ~ 310 μm; (h) The strain-stress curve of DN ionogels fabricated using zwitterionic microparticles with an average diameter of ~ 310 μm; (i) The combined strain-stress curve.


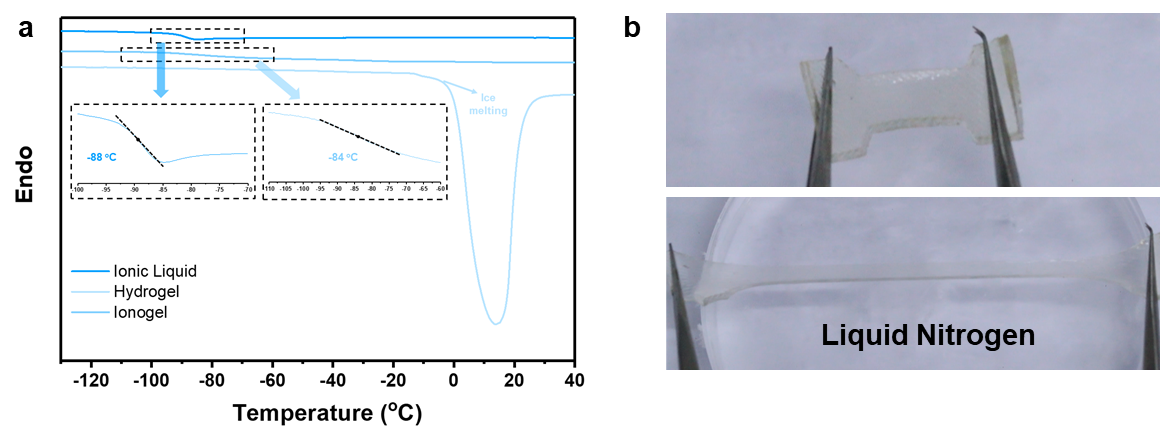


**Fig. S8.** (a) The DSC curve of native ionic liquid, PAAm-alginate DN hydrogel and DN ionogel. (b) The digital photo of stretching DN ionogel on top of liquid nitrogen.


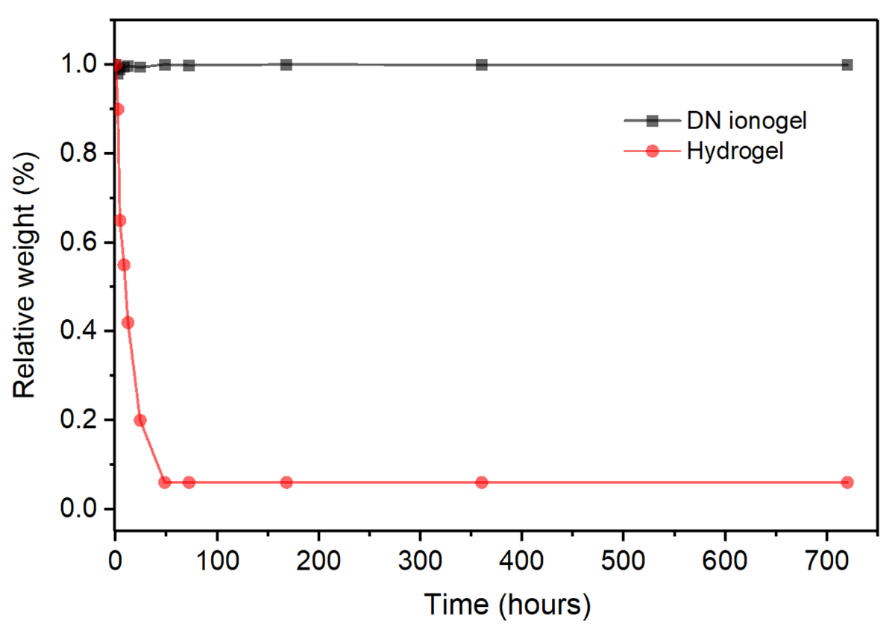


**Fig. S9.** Stability test of DN ionogel and alginate-polyacrylamide hydrogel for 3 month.


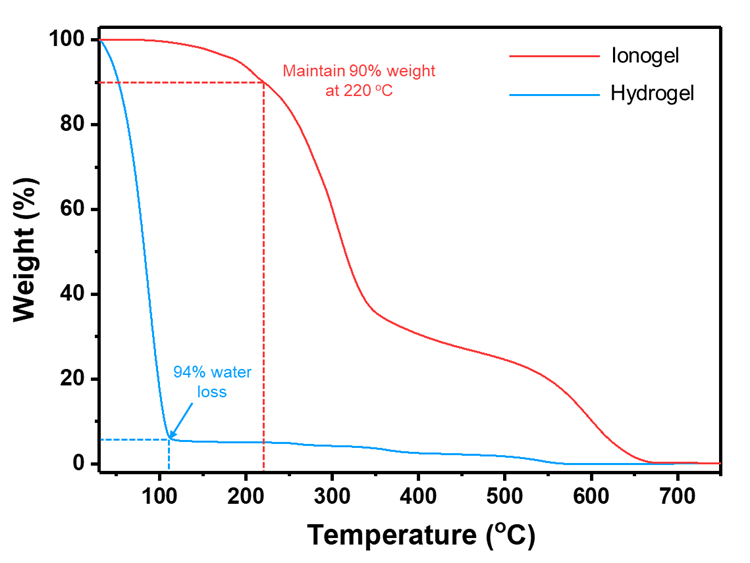


**Fig. S10.** The TGA curves of DN ionogel and DN hydrogel.


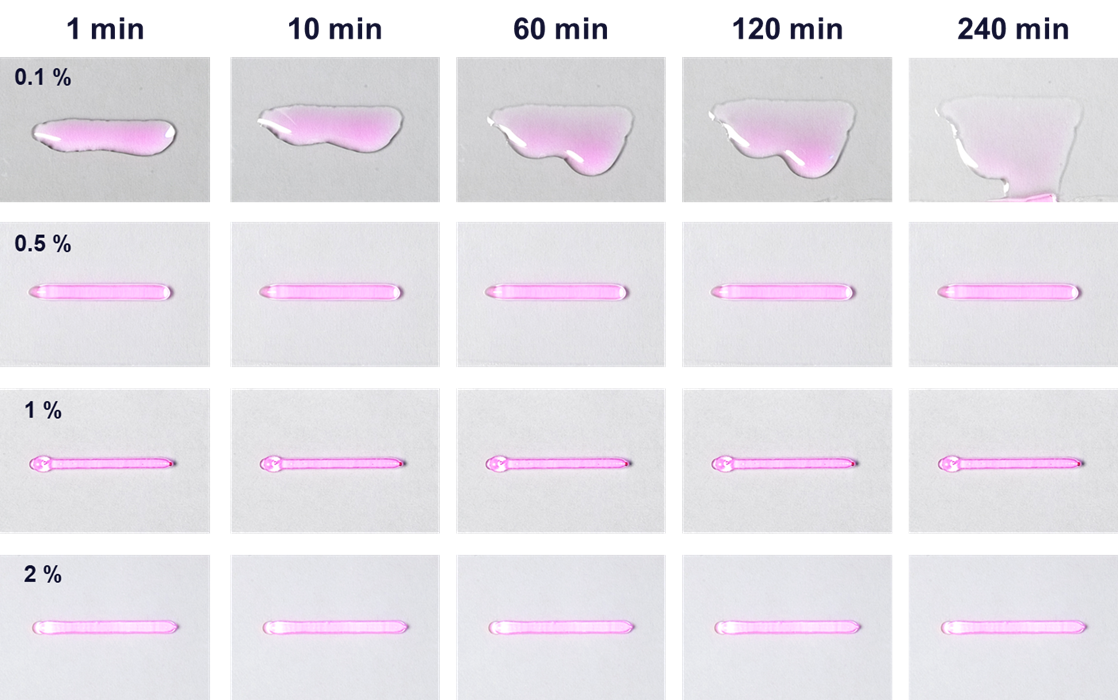


**Fig. S11.** The ionogel inks consisting of ZMPs with varied crosslinking density are printed on the substrate. The ionogel inks containing ZMPs with crosslinking density greater than 0.1% self-support on the substrate after standing vertically for 4 hours.


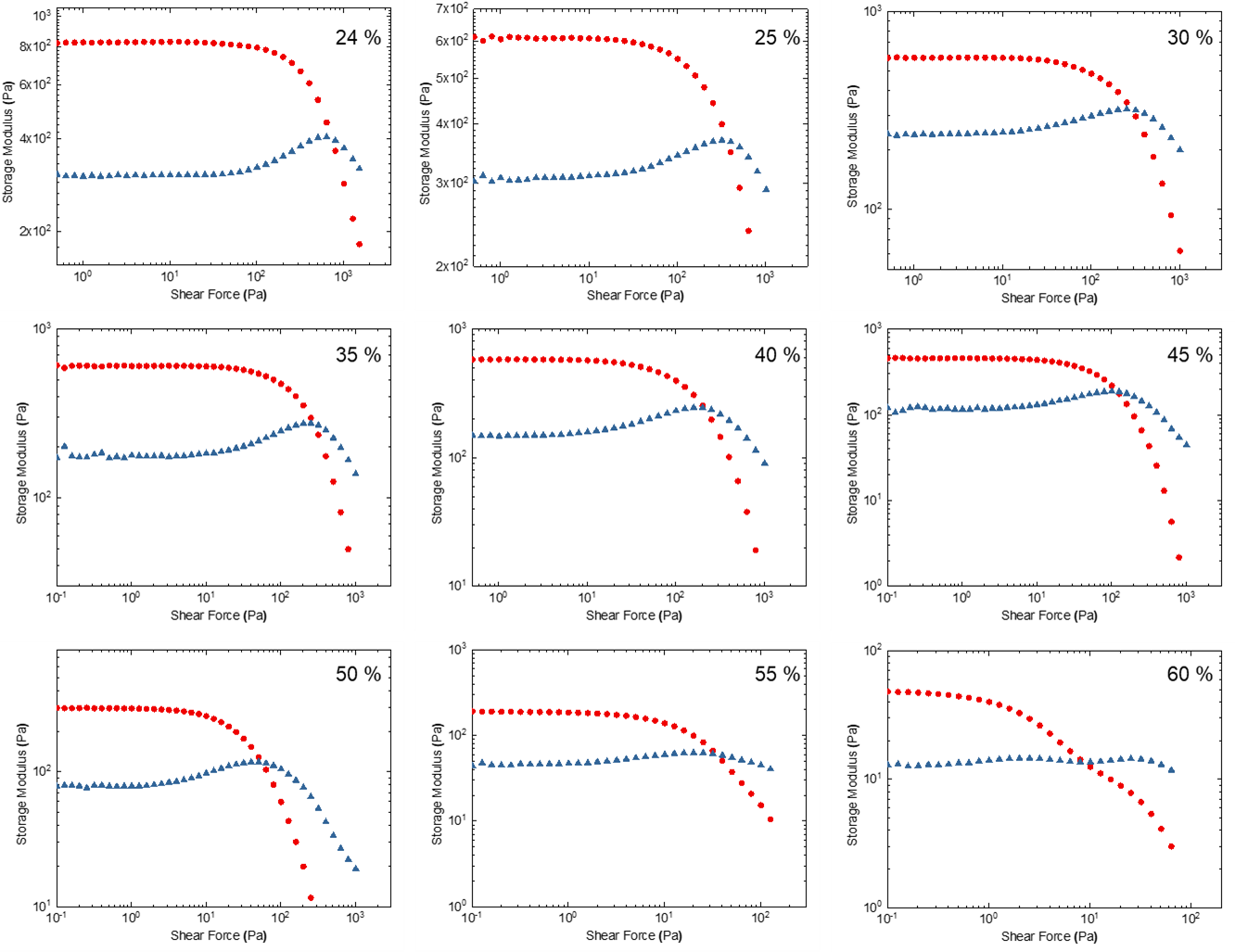


**Fig. S12.** Shear storage and loss modulus as a function of shear force for DN ionogel inks with different water content.


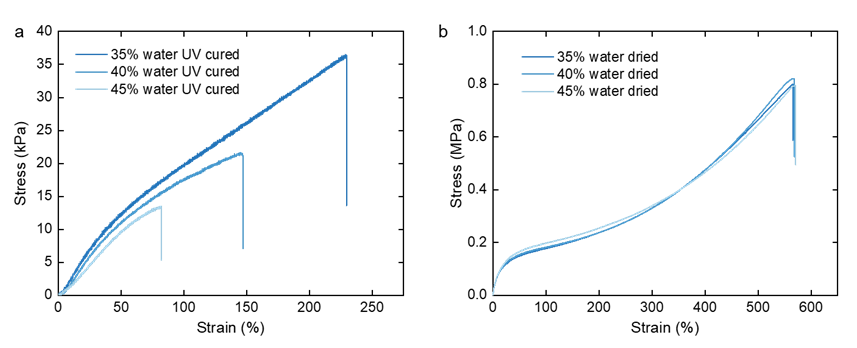


**Fig. S13.** (a) The strain-stress curves of DN ionogels specimens printed via DN ionogel inks with different water content (35%, 40% and 45%); (b) The strain-stress curves of DN ionogel specimens after dehydration.


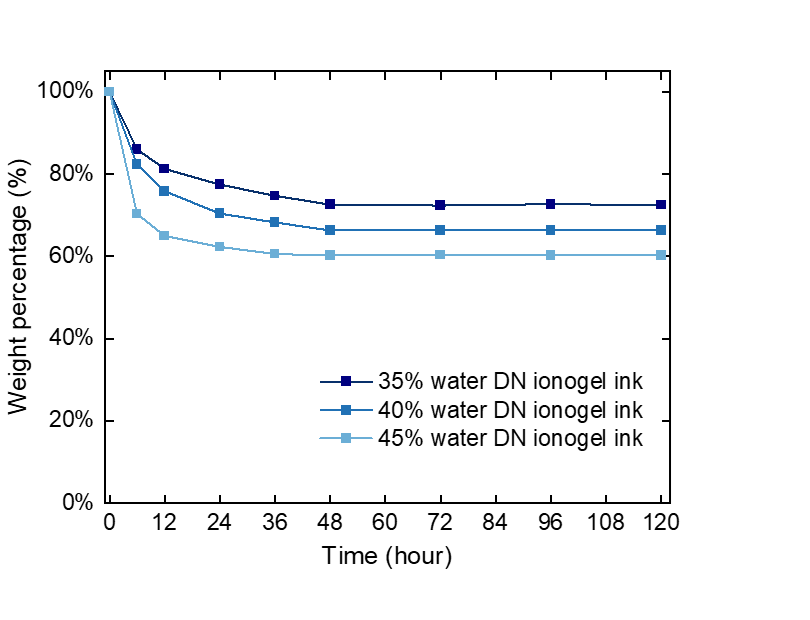


**Fig. S14.** The stability test of DN ionogel specimens printed via DN ionogel inks with different water content (35%, 40% and 45%).


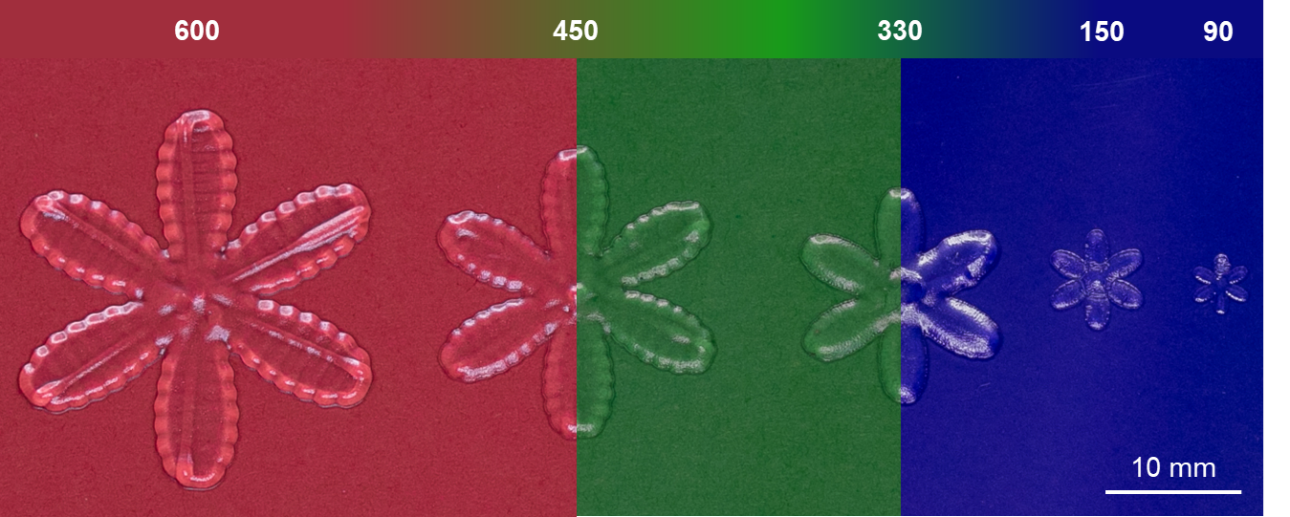


**Fig. S15.** Printing follower-shaped ionogels via nozzles with different diameters.


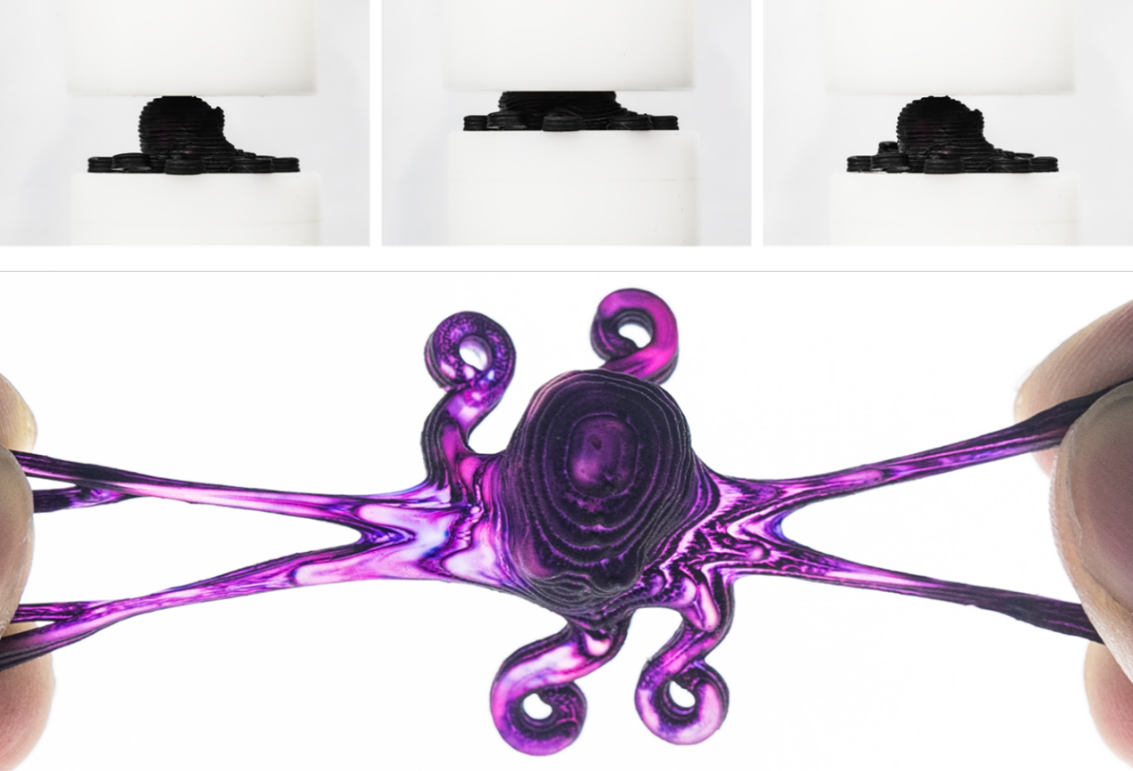


**Fig. S16.** Printed DN ionogel octopus under compression (top) and stretching (bottom).


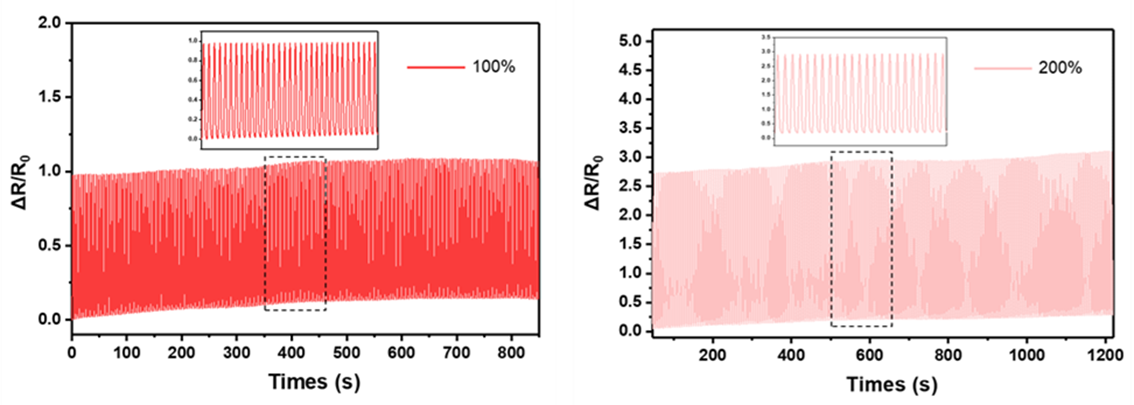


**Fig. S17.** The stability test of DN ionogel under 300 stretching and relaxation cycles at 100 % and 200 % strain.


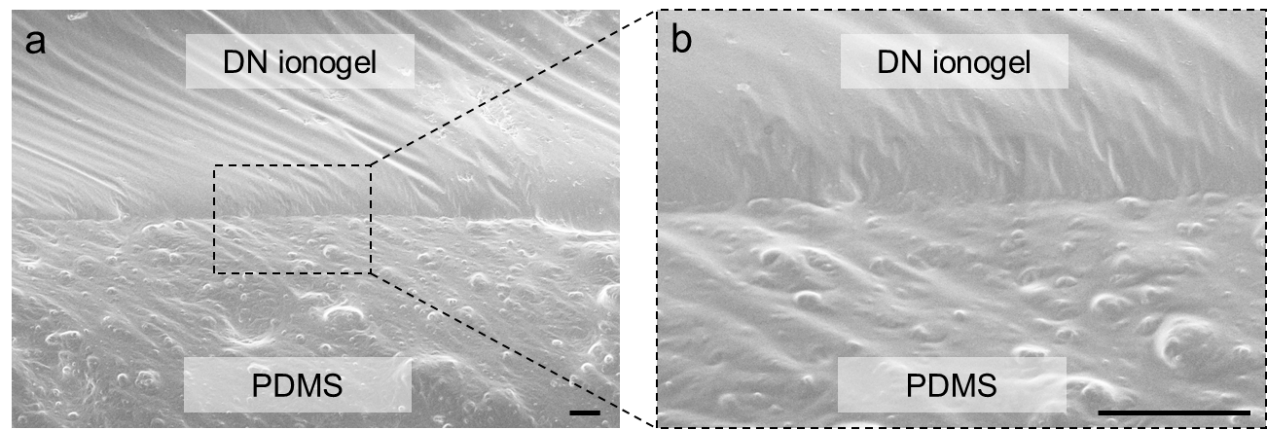


**Fig. S18.** (a) The SEM image of the morphology of the interface between DN ionogel and PDMS (scale bar = 10 μm); (b) The zoomed-in SEM image of microstructures at the interface between DN ionogel and PDMS (scale bar = 10 μm).


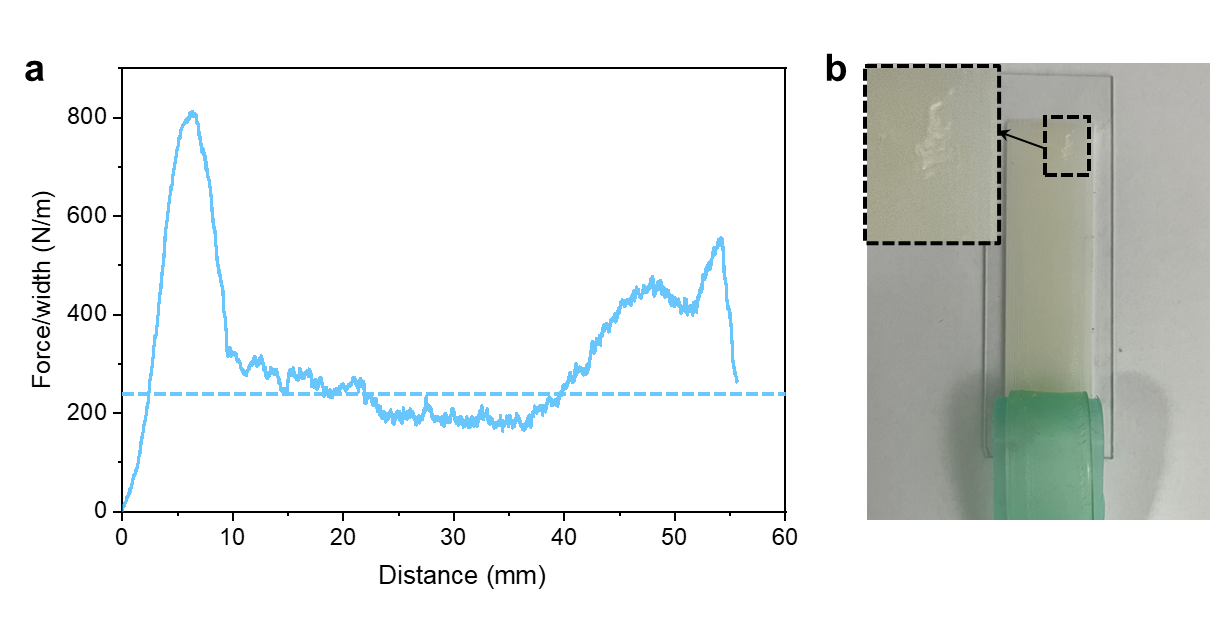


**Fig. S19.** (a) Curves of the peeling force per width of ionogel sheet versus displacement for ionogel-elastomer bonding. (b) Photo of ionogel-elastomer hybrid after 90^o^ peeling test, the residual ionogel is found on the elastomer surface (insert).


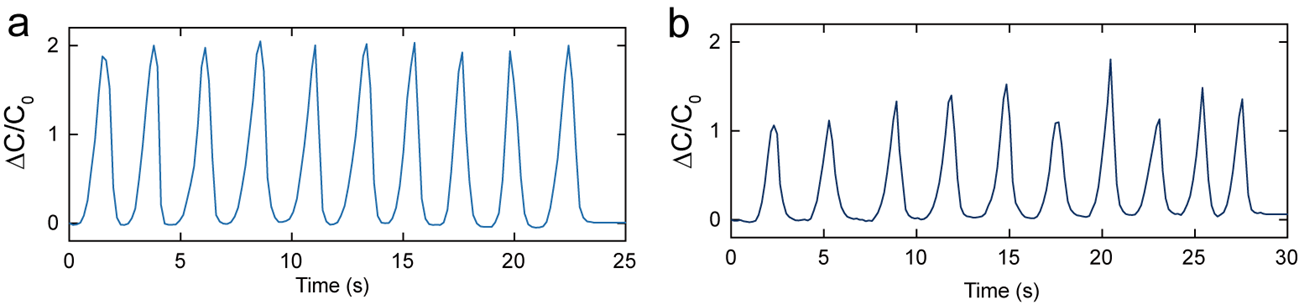


**Fig. S20.** (a) The DN ionogel-based capacitive ionic skin with covalent linkage between DN ionogels and PDMS in sensing bending-unbending of fingers; (b) The DN ionogel-based capacitive ionic skin without covalent linkage between DN ionogels and PDMS in sensing bending-unbending of fingers.


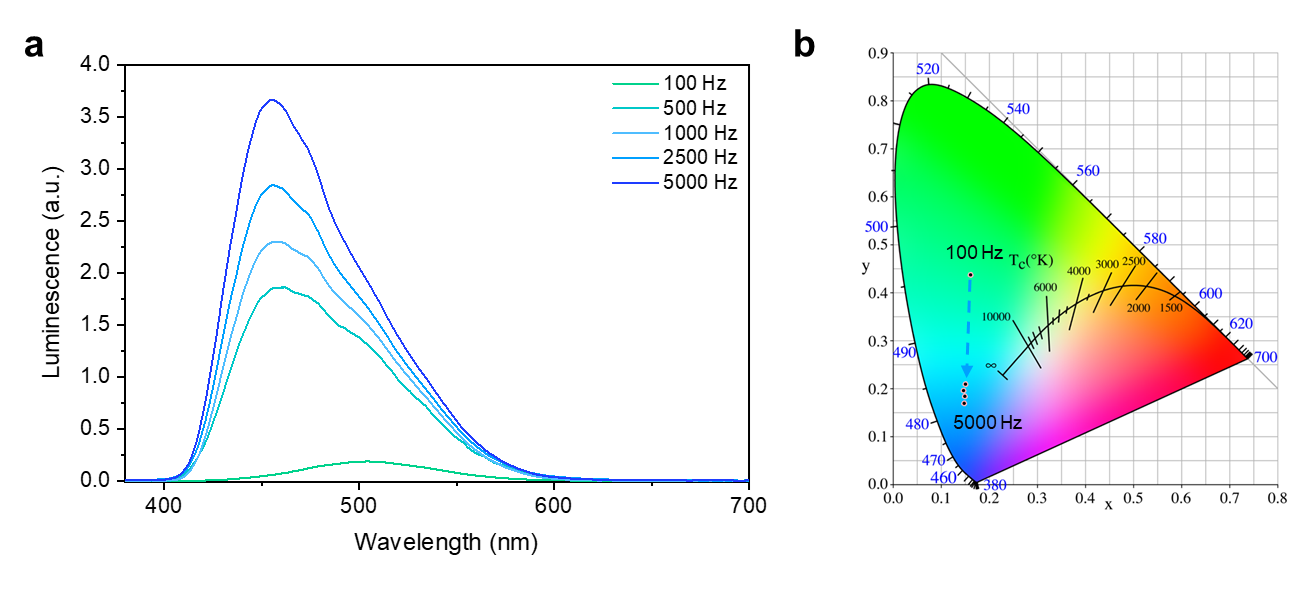


**Fig. S21.** (a) The electroluminescence spectra of ACEL device with a varying of applied frequency (100 Hz, 500 Hz, 1000 Hz, 2500 Hz and 5000 Hz). (b) The corresponding CIE1931 diagram of ACEL device during the change of applied frequency.
